# Global, Regional, and National Burden of Non-Rheumatic Valvular Heart Diseases in Women: A Systematic Analysis of Global Burden of Disease 1990–2021

**DOI:** 10.5334/gh.1422

**Published:** 2025-03-26

**Authors:** Liu Chenyu, Li Haochao, Chen Pengfei, Chen Mingjian, Zhao Diming, Wang Liqing

**Affiliations:** 1 Department of Cardiovascular Surgery, Fuwai Hospital, Chinese Academy of Medical Sciences & Peking Union Medical College/National Center for Cardiovascular Diseases, Beijing, China

**Keywords:** Global Burden of Disease database, non-rheumatic valvular heart diseases, women

## Abstract

**Background:**

The incidence of non-rheumatic valvular heart diseases (NRVHD) has shown an increasing trend. However, most studies have overlooked the impact of gender on the disease. Female patients, as a specific subgroup, have rarely been discussed independently. It is essential to conduct separate epidemiological studies to understand the latest epidemiological data for female NRVHD patients and to raise awareness among researchers and clinicians.

**Methods:**

Data from the Global Burden of Disease (GBD) 2021 database were retrieved to obtain epidemiological data on female NRVHD from both global and regional perspectives, covering 204 countries and territories. Joinpoint regression, age-period-cohort analysis, decomposition, and predictive analyses were employed to further examine the epidemiological data.

**Results:**

The incidence of female NRVHD patients has shown a continuous upward trend and is expected to persist in the future, particularly in regions with high and high-middle Socio-Demographic Index (SDI). However, in low and lower-middle SDI regions, patients experience relatively higher Disability-Adjusted Life Years (DALYs) and Years Lived with Disability (YLDs), with a greater number of heart failure cases attributed to NRVHD. Decomposition analysis indicates that the increase in the incidence of NRVHD and its subtypes is primarily driven by population growth and aging.

**Conclusions:**

With economic development and population aging, female NRVHD remains a significant healthcare burden for countries worldwide. Low- and middle-SDI regions should implement tertiary prevention strategies to address the impending shift in the spectrum of valvular heart diseases. Further clinical research should focus on female patients as a distinct subgroup of NRVHD, exploring the unique aspects of the disease in this population.

## Introduction

Valvular heart disease is one of the most common cardiac conditions. With the development of economic and healthcare conditions, the incidence of rheumatic heart disease has shown a declining trend across various regions globally (1). In contrast, the incidence of non-rheumatic valvular heart diseases (NRVHD) has significantly increased, with the most representative conditions being Non-rheumatic Calcific Aortic Valve Disease (NRAVD) and Non-rheumatic Degenerative Mitral Valve Disease (NRMVD). This rapid shift in the disease spectrum has posed numerous challenges to healthcare systems.

Male gender is considered one of the risk factors for NRVHD, and to date, women constitute a higher proportion of NRVHD patients. In 2019, patients with non-rheumatic valvular heart disease (NRVHD) were predominantly female, accounting for approximately 54% (2). However, the assessment of disease severity, staging, and the timing of surgery are mostly based on data derived from male patients (3). Female NRVHD patients represent a unique subgroup, yet they are rarely discussed independently. Applying diagnostic and therapeutic standards established for male to female patients may lead to significant errors. Li et al. conducted a detailed analysis of the incidence trends of Non-Rheumatic Valvular Heart Disease (NRVHD) using data from the Global Burden of Disease database (GBD) 2019. They also explored the risk factors for incidence and the attributable risks (2). However, their study did not specifically focus on the epidemiological characteristics of NRVHD among females. It is essential to treat female patients as a distinct subgroup within NRVHD and to discuss their unique characteristics separately.

GBD is currently the largest and most authoritative epidemiological statistics consortium, which includes over 600 billion highly standardized and comprehensive estimates measure health outcomes and systems, encompassing a comprehensive disease spectrum across 204 countries and regions. GBD regions are based on two criteria: epidemiological similarity and geographic closeness. Based on the patterns of causes of death, the world is divided into seven GBD super-regions, including Central Europe, Eastern Europe, and Central Asia; High-income; Latin America and the Caribbean; North Africa and the Middle East; South Asia; Southeast Asia, East Asia, and Oceania; and Sub-Saharan Africa (4). The GBD database provides comprehensive epidemiological data, contributing to 459 health outcomes and risk factors. These data allow for a comprehensive understanding of the global, regional, and national disease burden of female NRVHD, which is essential for the formulation and implementation of healthcare policies and for guiding scholars to pay special attention to the female patient population.

## Methods

The raw data for this study were obtained from the GBD study, which was publicly accessible after registration at https://www.healthdata.org/research-analysis/gbd-data (5,6). The data in the GBD database was derived from the aggregate data contributed by scholars worldwide. As all the summary data was de-identified, an informed consent or a relevant ethical review was unnecessary. In the GBD database, NRVHD primarily includes Non-Rheumatic Aortic Valve Disease (NRAVD), Non-Rheumatic Mitral Valve Disease (NRMVD), and other Non-Rheumatic Valvular Heart Diseases (ONRVHD). Due to incomplete data on ONRVHD, certain analyses excluded this group, focusing instead on the most representative categories, NRAVD and NRMVD.

After acquiring the raw data, comprehensive statistical analyses and graphical representations were conducted using the R language (version 4.4.1). The GBD study introduces the concept of the Socio-Demographic Index (SDI), which is calculated based on factors such as per capita income, education level, and fertility rate. This index categorizes countries into five levels—low, low-middle, middle, high-middle, and high, reflecting the average development level of a country or region. We accessed and utilized the latest data from the GBD 2021 database to analyze the trends in incidence, mortality, death and disability-adjusted life years (DALYs), years lived with disability (YLDs), and years of life lost (YLLs) for female NRVHD from 1990 to 2021. DALYs are a comprehensive indicator for measuring disease burden, representing the years of healthy life lost due to disease. YLLs measure the potential years of life lost due to premature death, while YLDs quantify non-fatal health losses caused by diseases or injuries (5). DALYs, YLLs, and YLDs collectively account for both premature mortality and disability caused by diseases, providing a holistic assessment of disease burden. In contrast, incidence and prevalence focus solely on the quantity or prevalence of diseases. DALYs integrate YLLs and YLDs to quantify the overall impact of diseases on population health and life expectancy, facilitating the comparison of relative burdens across different diseases or health issues. Compared with incidence and prevalence, these three indicators can assist public health policymakers in identifying priority areas for intervention and optimizing resource allocation (7). We also forecasted the incidence trends of NRVHD and its subtypes over the next 25 years and assessed the prevalence of heart failure caused by NRVHD.

### Joinpoint regression analysis

The Joinpoint regression model was employed to calculate the annual percent change (APC) and the average annual percent change (AAPC). APC is a measure used to describe the percentage change in a time series dataset over a specific period, assuming a constant rate of change. AAPC is a summary measure that provides an average percentage change over the entire study period. It is calculated by taking a weighted average of the APCs from different segments of a segmented regression model, providing a more comprehensive reflection of the overall trend. The formulas for APC and AAPC were detailed in the supplementary materials.

Model construction was conducted using Joinpoint software (version 5.1.0; National Cancer Institute, USA). Due to the non-normal distribution of most data, a log-linear regression model (ln y = xb) was selected for analysis.

### Age-period-cohort (APC) analysis

An APC analysis was conducted to assess the age effect and cohort effect on the burden of NRVHD in women. The data obtained from the GBD database were analyzed and visualized using the NIH online tool (https://analysistools.cancer.gov/apc/) (8). The age effect was displayed using Longitudinal and Cross-sectional Age Curves, while the cohort effect was presented using Period Relative Risk (RR). An RR > 1 indicated a higher relative risk of mortality/incidence compared to the reference period, whereas an RR < 1 indicated a lower relative risk.

### Decomposition Analysis

Decomposition analysis was applied to separate the epidemiological trends of NRVHD into three components: changes in age structure, population size, and epidemiological patterns. The specific formulas and computational principles for decomposition analysis are detailed in the study by Xie et al (9).

### Nordpred Prediction

Disease burden predictions, including incidence and YLDs of female NRVHD over the next 25 years, were performed using the Nordpred package (version 1.1) in R. An APC model with a generalized linear model (GLM) framework was utilized. Given that a power model with an exponent of five provided optimal results, the Nordpred package employed a fixed-exponent model with an exponent of five. The five-year data were disaggregated into annual estimates through the linear relationship within the model and analyzed accordingly.

### Gender difference analysis

To further explore the differences in the diagnosis and treatment outcomes of NRVHD between males and females in different SDI regions, we introduced the Age-Standardized Mortality-to-Incidence Ratio (ASMIR). This ratio is calculated by dividing the age-standardized mortality rate by the age-standardized incidence rate, and a line chart was used to illustrate its changes over the years.

## Results

From 1990 to 2021, the global incidence of NRVHD among women showed a marked increase (Table 1), rising from 391.87 thousand (95% UI: 357.55–430.82) in 1990 to 879.06 thousand (95% UI: 810.71–949.11) in 2021, with an Estimated Percentage Annual Change (EPAC) of 0.301 (95% UI: 0.230–0.372). High-SDI regions exhibited the highest EPAC, reaching 0.768 (95% UI: 0.685–0.852), whereas low-SDI regions showed a much lower EPAC of 0.145 (95% UI: 0.081–0.210). Additional details on mortality, YLDs, and YLLs related to NRVHD can be found in Supplementary Materials S1–S3. As economic development progresses, the age-standardized global mortality rate of NRVHD has shown a declining trend, with an Estimated Annual Percentage Change (EAPC) of –0.142 (95% UI: –0.246 to –0.037). However, the mortality rate in low-middle SDI regions has a slight upward trend, with an EAPC of 0.236 (95% UI: 0.172–0.300).

**Table 1:** Incidence, ASIR and EAPC in 1990-2021 among women with NRVHD.


LOCATION	1990		2021		1990–2021
		
	INCIDENCE (95% UI)	ASIR (95% UI)	INCIDENCE (95% UI)	ASIR (95% UI)	EAPC (95% UI)

**Global**	391.87 (357.55–430.82)	18.33 (16.77–20.16)	879.06 (810.71–949.11)	18.93 (17.45–20.32)	0.30 (0.23–0.37)

**SDI**					

**Low SDI**	4.08 (3.07–4.46)	3.63 (3.32–3.90)	9.97 (9.11–10.84)	3.83 (3.55–4.13)	0.14 (0.08–0.21)

**Low-middle SDI**	16.27 (14.70–17.85)	5.31 (4.93–5.94)	44.83 (40.55–49.08)	5.93 (5.42–6.45)	0.31 (0.25–0.37)

**Middle SDI**	37.33 (34.23–40.61)	6.86 (6.37–7.46)	119.08 (108.75–129.79)	8.22 (7.53–8.98)	0.73 (0.66–0.80)

**High-middle SDI**	104.48 (95.88–114.75)	18.19 (16.60–19.93)	226.37 (209.17–243.78)	20.42 (19.02–22.02)	0.62 (0.51–0.72)

**High SDI**	229.24 (207.13–252.64)	36.00 (32.79–39.62)	477.88 (438.41–518.49)	42.83 (39.55–46.81)	0.77 (0.69–0.85)

**GBD Region**					

**Andean Latin Americ**a	0.57 (0.50–0.66)	5.15 (4.43–5.97)	2.48 (2.17–2.81)	7.91 (6.92–9.08)	1.34 (1.31–1.38)

**Australasia**	3.58 (3.13–4.05)	26.45 (23.21–29.67)	9.56 (8.36–10.77)	33.02 (28.83–36.94)	0.80 (0.76–0.84)

**Caribbean**	0.84 (0.74–0.95)	6.10 (5.43–6.99)	2.26 (1.99–2.54)	8.02 (7.01–9.08)	0.89 (0.84–0.95)

**Central Asia**	5.36 (4.45–6.42)	19.12 (16.02–22.83)	12.71 (10.97–14.61)	26.13 (22.52–30.28)	1.34 (1.13–1.55)

**Central Europe**	20.76 (18.21–23.40)	24.02 (21.24–27.02)	41.74 (37.56–46.02)	35.73 (32.24–39.69)	1.68 (1.39–1.96)

**Central Latin America**	3.54 (3.06–4.05)	7.81 (6.73–8.95)	14.59 (12.48–16.76)	10.64 (9.13–12.25)	1.28 (1.15–1.42)

**Central Sub-Saharan Africa**	0.36 (0.32–0.40)	3.02 (2.73–3.34)	0.90 (0.80–1.01)	3.02 (2.74–3.38)	–0.01 (–0.09–0.07)

**East Asia**	33.49 (31.16–36.07)	7.24 (6.72–7.84)	105.98 (99.29–113.43)	8.83 (8.29–9.47)	0.81 (0.73–0.90)

**Eastern Europe**	32.63 (29.33–36.37)	18.02 (16.23–19.92)	55.71 (49.32–63.11)	25.63 (22.96–28.97)	1.45 (1.29–1.61)

**Eastern Sub-Saharan Africa**	1.15 (1.03–1.27)	3.02 (2.73–3.34)	2.56 (2.32–2.83)	2.87 (2.52–3.13)	–0.27 (–0.35––0.19)

**High-income Asia Pacific**	43.80 (39.06–49.06)	37.82 (33.81–42.25)	95.11 (85.13–106.51)	41.65 (37.73–46.17)	0.46 (0.37–0.55)

**High-income North America**	102.74 (91.42–114.65)	51.13 (46.15–56.66)	211.56 (194.36–229.26)	58.12 (53.57–62.65)	0.59 (0.53–0.66)

**North Africa and Middle East**	7.62 (6.87–8.48)	8.52 (7.73–9.54)	21.70 (19.55–24.09)	9.62 (8.32–10.16)	0.36 (0.28–0.44)

**Oceania**	0.08 (0.07–0.09)	5.35 (4.53–6.45)	0.20 (0.17–0.24)	5.73 (4.96–6.73)	0.14 (0.11–0.17)

**South Asia**	12.82 (11.78–13.89)	4.83 (4.43–5.24)	38.76 (35.61–42.22)	5.13 (4.75–5.66)	0.22 (0.13–0.31)

**Southeast Asia**	7.44 (6.60–8.43)	5.53 (4.95–6.23)	24.12 (21.46–27.31)	6.72 (6.00–7.59)	0.60 (0.53–0.67)

**Southern Latin America**	4.88 (4.30–5.57)	18.85 (16.73–21.46)	13.18 (11.52–15.25)	26.83 (23.55–30.98)	1.07 (0.87–1.27)

**Southern Sub-Saharan Africa**	0.49 (0.44–0.54)	3.03 (2.75–3.32)	1.03 (0.91–1.15)	3.13 (2.83–3.42)	–0.12 (–0.20–0.05)

**Tropical Latin America**	5.10 (4.52–5.79)	9.94 (8.86–11.34)	16.95 (14.58–19.59)	11.93 (10.34–13.82)	0.62 (0.51–0.72)

**Western Europe**	103.63 (94.11–113.83)	30.93 (28.53–33.76)	205.43 (189.31–221.37)	42.73 (39.66–45.98)	1.20 (1.04–1.37)

**Western Sub-Saharan Africa**	0.96 (0.87–1.06)	2.13 (1.95–2.33)	2.56 (2.29–2.81)	2.24 (2.03–2.46)	0.20 (0.18–0.22)


* The unit of incidence is thousand people. ASIR, age-standardized incidence rate; EAPC, Estimated Annual Percentage Change; 95%UI, 95% Uncertainty Interval; NRVHD, non-rheumatic valvular heart diseases; SDI, Socio-demographic Index.

### Joinpoint model

**Figure 1:**
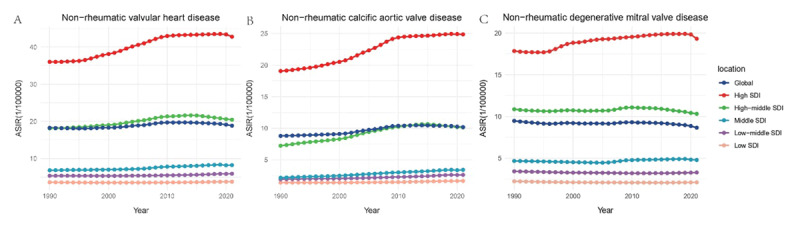
Joinpoint regression analysis for NRVHD in women. * Each point represents the average annual percent change for each year; the color of the points represents the global situation compared to different Socio-Demographic Index regions.

Using the Joinpoint model, we constructed the annual AAPC for different SDI regions (Figure 1, Supplementary Materials S5). Four or five breakpoints were selected for each SDI region, with all breakpoints showing p values < 0.0001, indicating that the differences in AAPC compared to 0 were statistically significant.

From 1990 to 2021, the global AAPC for female NRVHD was consistently positive. High-SDI and middle-high SDI regions displayed similar AAPC trends, with a rapid increase from 1995 to 2010, followed by stabilization. Conversely, low, low-middle, and middle SDI regions exhibited stable but slow growth, with much lower rates of increase. On a global scale, the AAPC for NRVHD appeared to stabilize after 2010. The AAPC curve for NRAVD closely mirrored that of NRVHD, while NRMVD showed a slight downward trend.

**Figure 2:**
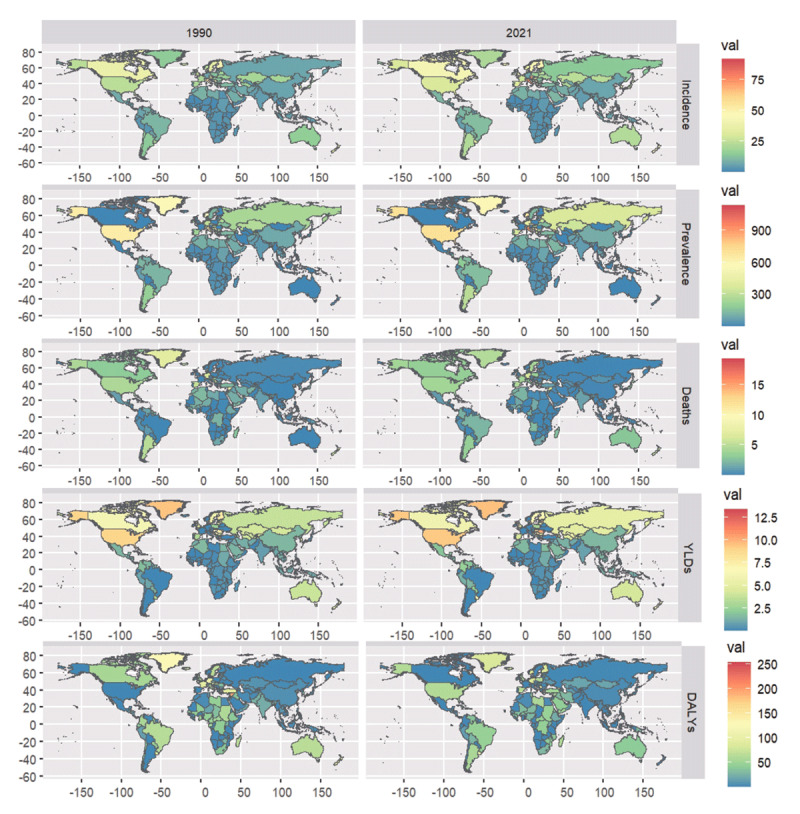
Changes in the epidemiological characteristics of NRVHD among women in 204 countries and regions from 1990 to 2021.

The incidence, prevalence, deaths, YLDs, and DALYs for NRVHD in 204 countries were visualized on the world map (Figure 2), with specific data available in Supplementary Materials S4. Consistent with the SDI classification results, the disease burden of female NRVHD increased significantly in developed regions. For example, in the United States, the incidence rose from 95.75 thousand (95% UI: 85.28–106.81) to 195.16 thousand (95% UI: 178.66–212.47), reflecting an increase of 103.8%. Meanwhile, China, as a representative of middle-SDI countries experiencing rapid economic growth and population aging, showed a rise in incidence from 0.32 (95% UI 0.30–0.34) to 1.01 (95% UI 0.95–1.08), a growth of 217.1%. Similarly, DALYs in the United States increased from 162.56 thousand (95% UI 145.10–178.32) to 232.32 thousand (95% UI 196.31–260.87), representing a 42.9% increase. In China, DALYs rose from 25.00 thousand (95% UI 15.77–33.61) to 37.09 thousand (95% UI 26.91–52.18), an increase of 48.4%.

### APC model

**Figure 3:**
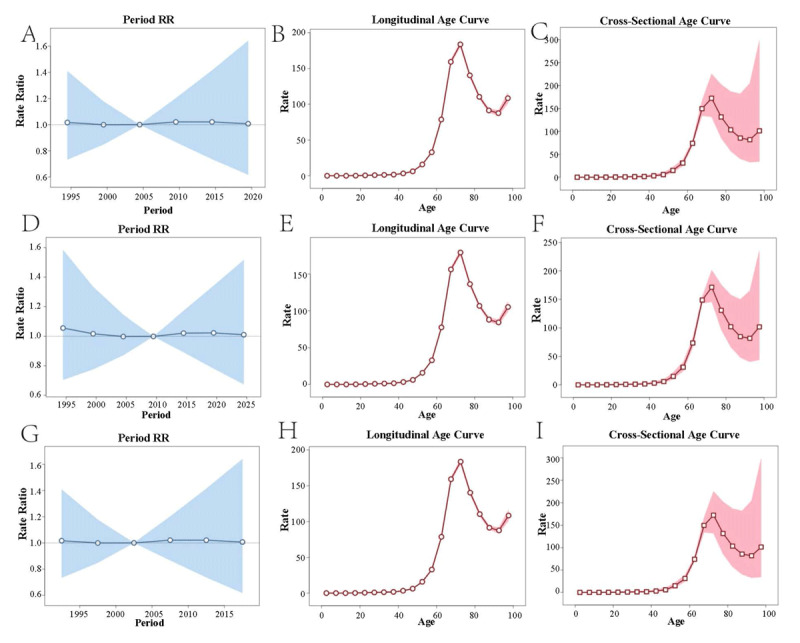
APC model results for NRVHD and its subtype. * Figure ABC represents the Annual Percent Change results for NRVHD. Figure DEF represents the APC results for NRAVD. Figure GHI represents the APC results for NRMVD. Figure ADG illustrates period effects, reflecting the risk ratios compared to a reference year. Figure BEH shows Longitudinal Age Curves, which depict the longitudinal distribution characteristics of the disease by age. Figure CFI reflects the cross-sectional age distribution observed annually in each study.

The results of the APC model were shown in Figure 3. Interestingly, NRVHD and its subtypes exhibit similar patterns in the APC model. Figures A, D, and G reveal that the global average incidence of NRVHD has undergone relatively small changes over time, with a slight decline followed by a gradual increase. Figures B, E, and H show that among female NRVHD patients, the incidence within the same birth cohort follows a U-shaped curve, peaking at age 75 before sharply declining. The cross-sectional age curve for each year resembles the longitudinal age curve, albeit with relatively lower volatility.

### Decomposition analysis

**Figure 4:**
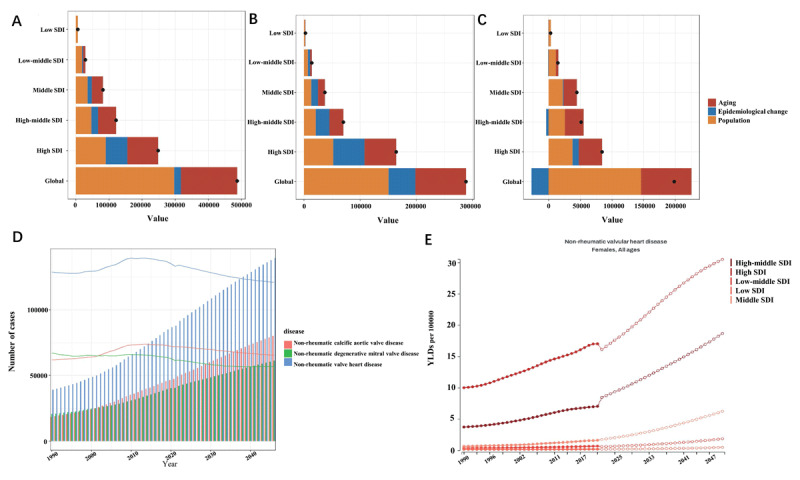
Decomposition Analysis and Future Prediction Models in Female Patients with NRVHD. * Figure ABC presents the decomposition analysis results, where Figure A represents NRVHD, Figure B represents NRAVD, and Figure C represents NRMVD. The black dots in the figures represent the results of the three influencing factors. Figure DE shows the prediction analysis results: Figure D reflects the incidence rates of NRVHD and its subtypes up to 2046, with lines representing the changes in age-standardized incidence rates. Figure E displays the predicted future trends of total NRVHD occurrences across different Socio-Demographic Index regions.

The decomposition analysis indicates that the continuous rise in NRVHD incidence among females (Figure 4A-C) was primarily driven by population growth. Interestingly, as SDI levels increase, epidemiological shifts and population aging contribute more significantly to the growth in incidence.

For NRAVD, the epidemiological decomposition characteristics are similar to those of overall NRVHD. In contrast, for NRMVD, population aging and rising SDI levels also contribute to the increased incidence, but the proportion attributed to epidemiological changes is relatively small. From a global perspective, the contribution of epidemiological changes to NRMVD even demonstrates a certain degree of negative impact.

### Nordpred prediction

Figure 4D presented the disease incidence forecast. It is expected that as the global population grows and aging intensifies, the incidence of NRVHD and its subtypes will continue to rise. However, age-standardized incidence rates are slightly declining and are expected to stabilize afterward. Figure 4E showed a rapid increase in YLDs in high SDI and high-middle SDI regions, with middle SDI regions also showing an upward trend. In contrast, in low and low-middle SDI regions, the rate of increase in YLDs due to NRVHD in women is expected to be slower over the next 25 years.

Finally, we assessed the incidence of heart failure caused by NRVHD (Table 2). On a global scale, YLDs and prevalence related to heart failure due to NRVHD have shown a decreasing trend from 1990 to 2021 (3.56 vs 2.63 in YLDs, 39.74 vs 29.45 in prevalence). In high SDI regions, heart failure due to NRVHD also shows a decreasing trend (5.44 vs 4.13 in YLDs, 60.52 vs 45.92 in prevalence), while in low, low-middle, and middle SDI regions, the YLDs and prevalence related to heart failure show the opposite increasing trend. However, for the time being, higher SDI regions still carry a higher disease burden from NRVHD-related heart failure in female patients.

**Table 2:** Heart failure caused by non-rheumatic valvular heart disease. * YLDs, age-standardized incidence rate; SDI, Socio-demographic Index.


LOCATION	1990		2021	
	
	YLDS	PREVALENCE	YLDS	PREVALENCE

**Global**	3.56 (2.04–6.06)	39.74 (26.61–61.51)	2.63 (1.49–4.51)	29.45 (19.03–45.44)

**SDI**				

**Low SDI**	0.55 (0.30–1.00)	6.23 (4.11–9.78)	0.60 (0.32–1.06)	6.76 (4.49–10.45)

**Low-middle SDI**	1.03 (0.57–1.78)	11.64 (7.71–18.24)	1.12 (0.62–1.96)	12.67 (8.44–19.55)

**Middle SDI**	1.45 (0.81–2.50)	16.40 (10.74–25.22)	1.49 (0.84–2.54)	16.73 (11.17–25.72)

**High-middle SDI**	3.71 (2.08–6.37)	41.50 (27.62–64.49)	2.98 (1.70–5.09)	33.35 (21.52–51.61)

**High SDI**	5.44 (3.09–9.36)	60.52 (39.74–94.59)	4.13 (2.26–7.11)	45.92 (28.69–73.03)


### Gender difference analysis

The results of the analysis were presented in Supplementary Material S10. From 1990 to 2021, in different SDI regions, the ASMIR of female NRVHD patients was consistently higher than that of males. In 1990, the ASMIR for males was 0.085, while 0.146 for females. In 2021, the ASMIR for males remained at 0.073, and 0.120 for females. The ASMIR in High SDI regions was significantly lower than that in Low SDI regions. These findings reflect that the level of diagnosis and treatment for female NRVHD patients was considerably lower than that for males, especially in economically underdeveloped regions.

## Discussion

Valvular heart disease is the most common cause of heart failure in female patients, and compared to males, female patients have a higher incidence of major adverse cardiovascular events (MACE) (10). Among patients with NRAVD, female patients tend to be older on average, have more severe aortic stenosis, smaller valve sizes, and a significantly increased risk of prosthesis-patient mismatch, resulting in worse outcomes following aortic valve replacement surgery compared to male patients (11,12). In patients with aortic regurgitation, clinical guidelines are primarily based on male patients, and there is a lack of high-quality evidence regarding the relationship between left ventricular dimensions and surgical indications in female patients (13). In mitral valve disease, females tend to develop symptoms later despite having the same degree of valve involvement as males, with lower consultation rates and more severe conditions at presentation (14). Compared to male patients, female patients experience poorer short-term surgical outcomes. They are more likely to undergo mitral valve replacement rather than repair, which directly contributes to a worse long-term prognosis for female patients (15,16,17).

Through a comprehensive analysis of the GBD database from 1990 to 2021, we have obtained detailed epidemiological information on female NRVHD patients globally, regionally, and nationally. Additionally, we have projected the incidence trends for the next 25 years. NRVHD remains one of the most impactful diseases among cardiovascular diseases. With the aging population and the increase in population size, the disease burden of NRVHD on female patients is expected to intensify. We predicted that by 2046, the global incidence of NRVHD in women will increase to 1395.48 thousand, an approximate increase of 58.7% compared to 2021.

The increase in the number of female NRVHD patients in developed regions is primarily driven by the aging of the population, whereas in less developed regions, it is mainly due to the overall population growth. The results of the decomposition analysis have confirmed this view (18). In high-SDI regions, the increase in NRVHD incidence among female patients from 1990 to 2021 can primarily be attributed to demographic factors, such as population growth and the increase in the average age. Changes in the epidemiological disease spectrum contributed to the rise in the incidence of NRVHD and NRAVD, while a negative contribution in NRMVD, possibly related to the decline in the incidence and DALYs of ischemic heart disease in many countries (19), particularly in developed nations, which in turn may have led to a decrease in the incidence of mitral valve disease (20).

The incidence of NRVHD in females was on an upward trend, although this increase was gradually decelerating, the total number of affected individuals continues to rise. The APC model constructed using Joinpoint predicts the trend of the AAPC. In high SDI and high-middle SDI regions, the AAPC increased rapidly between 1995 and 2015, after which the rate of increase flattened. However, in other SDI regions, the incidence continues to rise slowly. It is foreseeable that in these low-SDI regions, the growth rate of NRVHD incidence in female patients will continue to increase over the next period. This trend has been confirmed in our predictive analysis, particularly in the low-middle SDI regions, where the slope of the growth curve is slowly increasing, indicating an acceleration in the incidence growth rate.

From the epidemiological burden data across different countries and regions, although high-SDI countries have a higher overall incidence, the death rates, YLDs, and DALYs were on average lower than in low-SDI countries. This suggests that high-SDI countries pay more attention to female NRVHD and have more effective prevention and treatment strategies in place. For example, in India, the incidence of NRVHD in women in 2021 was 311.68 thousand, with 6.89 thousand deaths and 158.56 thousand DALYs. In contrast, in high-SDI countries like the United Kingdom, the incidence was 26.11 thousand, with 3.83 thousand deaths and only 50.04 thousand DALYs. Consistent with the findings of Nejad et al., the global healthcare quality for NRVHD is generally acceptable; however, patients in regions with low SDI levels should receive greater attention (21). Heart failure was the primary initial symptom of valvular disease, and this symptom was more commonly observed in female patients due to delayed disease diagnosis (22,23). The incidence of heart failure caused by NRVHD in female patients showed a declining trend in the high-middle SDI and high SDI regions from 1990 to 2021, while in other SDI regions, this figure significantly increased. This reflected the better healthcare system and treatment capabilities for female NRVHD patients in higher SDI regions.

Our study, based on the Global Burden of Disease (GBD) database, highlights the significant disease burden of NRVHD in women. These findings suggest several avenues for future research. For example, the observed high incidence and prevalence of NRVHD in women warrant further investigation into the underlying biological and sociodemographic factors contributing to these disparities. Future studies could explore the role of sex hormones, genetic predispositions, and lifestyle factors in the pathogenesis of NRVHD. Additionally, our analysis could serve as a foundation for longitudinal cohort studies to better understand the long-term outcomes and risk factors unique to women with NRVHD. The disease burden in women, particularly in older age groups, underscores the need for targeted screening and early intervention strategies. Furthermore, our study highlights the importance of addressing sex-specific differences in clinical guidelines for the management of valvular heart diseases, potentially improving patient outcomes through more personalized care. Therefore, we call for the establishment of a tiered prevention system for NRVHD in low and low-middle SDI countries to reduce the disease burden and its impact on survival rates, especially among female patients. In the foreseeable future, these regions will be significantly affected by the migration of the NRVHD disease spectrum and will bear a substantial health and economic burden. Fortunately, there has been growing attention from scholars toward female valvular heart diseases, with some randomized controlled trials now underway (24). However, the enrollment rate of female patients remains insufficient, and our study may help attract more scholarly attention to this issue.

This study has several limitations. First, countries from low or low-to-middle SDI regions may have deficiencies in the accuracy and timeliness of reporting epidemiological data, which could compromise the reliability of some of the results. Second, the increased incidence of certain diseases can be partly attributed to advancements in diagnostic equipment and techniques, leading to the identification of previously undiagnosed cases. Third, the absence of patient data on ONRVHD may limit the comprehensiveness of the study findings, precluding additional benefits for patients with other rare valvular heart diseases.

## Conclusion

This study utilized data from the GBD database from 1990 to 2021 to assess the epidemiological characteristics of female NRVHD patients. With the global population increasing, the number of NRVHD patients is expected to rise significantly. The disease burden in high-SDI regions is notably higher than in low-SDI regions, but these areas have lower mortality rates and YLDs. Low-SDI regions should adopt proactive health policies to address the shifting disease spectrum.

## Additional File

The additional file for this article can be found as follows:

10.5334/gh.1422.s1 Supplementary Materials. *Review search terms*.

## References

[1] Ruan R, et al. Global, Regional, and National Advances Toward the Management of Rheumatic Heart Disease Based on the Global Burden of Disease Study 2019. J Am Heart Assoc. 2023;12:e028921. DOI: 10.1161/JAHA.122.028921 37366108 PMC10356074

[2] Li L, et al. Global, Regional, and National Burden of Nonrheumatic Valvular Heart Disease and Its Attributable Risk Factors in 204 Countries and Territories, 1990 to 2019: Results From the Global Burden of Disease Study 2019. J Am Heart Assoc. 2024;13:e034459. DOI: 10.1161/JAHA.124.034459 39424422 PMC11935725

[3] Fleury MA, Clavel MA. Sex and Race Differences in the Pathophysiology, Diagnosis, Treatment, and Outcomes of Valvular Heart Diseases. Can J Cardiol. 2021;37:980–991. DOI: 10.1016/j.cjca.2021.02.003 33581193

[4] Liang X, Lyu Y, Li J, Li Y, Chi C. Global, regional, and national burden of preterm birth, 1990–2021: a systematic analysis from the global burden of disease study 2021. E Clinical Medicine. 2024;76:102840. DOI: 10.1016/j.eclinm.2024.102840 PMC11462015 39386159

[5] GBD 2021 Diseases and Injuries Collaborators. Global incidence, prevalence, years lived with disability (YLDs), disability-adjusted life-years (DALYs), and healthy life expectancy (HALE) for 371 diseases and injuries in 204 countries and territories and 811 subnational locations, 1990–2021: a systematic analysis for the Global Burden of Disease Study 2021. Lancet. 2024;403:2133–2161. DOI: 10.1016/S0140-6736(24)00757-8 38642570 PMC11122111

[6] GBD 2021 Diseases and Injuries Collaborators. Global burden of 288 causes of death and life expectancy decomposition in 204 countries and territories and 811 subnational locations, 1990–2021: a systematic analysis for the Global Burden of Disease Study 2021. Lancet. 2024;403:2100–2132. DOI: 10.1016/S0140-6736(24)00367-2 38582094 PMC11126520

[7] Augustovski F, et al. Measuring the Benefits of Healthcare: DALYs and QALYs – Does the Choice of Measure Matter? A Case Study of Two Preventive Interventions. Int J Health Policy Manag. 2018;7:120–136. DOI: 10.15171/ijhpm.2017.47 29524936 PMC5819372

[8] Rosenberg PS, Check DP, Anderson WF. A web tool for age-period-cohort analysis of cancer incidence and mortality rates. Cancer Epidemiol Biomarkers Prev. 2014;23:2296–2302. DOI: 10.1158/1055-9965.EPI-14-0300 25146089 PMC4221491

[9] Xie Y, et al. Analysis of the Global Burden of Disease study highlights the global, regional, and national trends of chronic kidney disease epidemiology from 1990 to 2016. Kidney Int. 2018;94:567–581. DOI: 10.1016/j.kint.2018.04.011 30078514

[10] Nitsche C, Koschutnik M, Kammerlander A, Hengstenberg C, Mascherbauer J. Gender-specific differences in valvular heart disease. Wien Klin Wochenschr. 2020;132:61–68. DOI: 10.1007/s00508-019-01603-x 31997064 PMC7035223

[11] Onorati F, et al. Different impact of sex on baseline characteristics and major periprocedural outcomes of transcatheter and surgical aortic valve interventions: Results of the multicenter Italian OBSERVANT Registry. J Thorac Cardiovasc Surg. 2014;147:1529–1539. DOI: 10.1016/j.jtcvs.2013.05.039 23856202

[12] Youssef G. Valvular heart diseases in women. Egypt Heart J. 2021;73:58. DOI: 10.1186/s43044-021-00184-3 34176027 PMC8236007

[13] Malahfji M, Saeed M, Zoghbi WA. Aortic Regurgitation: Review of the Diagnostic Criteria and the Management Guidelines. Curr Cardiol Rep. 2023;25:1373–1380. DOI: 10.1007/s11886-023-01955-x 37715804

[14] Nkomo VT, et al. Burden of valvular heart diseases: a population-based study. Lancet. 2006;368:1005–1011. DOI: 10.1016/S0140-6736(06)69208-8 16980116

[15] Vassileva CM, et al. Gender differences in long-term survival of Medicare beneficiaries undergoing mitral valve operations. Ann Thorac Surg. 2013;96:1367–1373. DOI: 10.1016/j.athoracsur.2013.04.055 23915585

[16] Chang FC, et al. Sex Differences in Epidemiological Distribution and Outcomes of Surgical Mitral Valve Disease. Circ J. 2024;88:579–588. DOI: 10.1253/circj.CJ-23-0687 38267036

[17] Tran A, Ruel M, Chan V. Gender differences in outcomes following cardiac surgery: implications for managing patients with mitral valve disease. Curr Opin Cardiol. 2015;30:151–154. DOI: 10.1097/HCO.0000000000000150 25574891

[18] Coffey S, et al. Global epidemiology of valvular heart disease. Nat Rev Cardiol. 2021;18:853–864. DOI: 10.1038/s41569-021-00570-z 34172950

[19] Safiri S, et al. Burden of ischemic heart disease and its attributable risk factors in 204 countries and territories, 1990–2019. Eur J Prev Cardiol. 2022;29:420–431. DOI: 10.1093/eurjpc/zwab213 34922374

[20] Enriquez-Sarano M, Akins CW, Vahanian A. Mitral regurgitation. Lancet. 2009;373:1382–1394. DOI: 10.1016/S0140-6736(09)60692-9 19356795

[21] Nejad M, et al. Global and regional burden and quality of care of non-rheumatic valvular heart diseases: a systematic analysis of Global Burden of Disease 1990–2017. Int J Qual Health Care. 2022;34. DOI: 10.1093/intqhc/mzac026 35434737

[22] Welt FGP, Fang JC. Pressure Volume System for Management of Heart Failure and Valvular Heart Disease. Curr Cardiol Rep. 2019;21:153. DOI: 10.1007/s11886-019-1247-0 31768659

[23] Crousillat DR, Wood MJ. Valvular Heart Disease and Heart Failure in Women. Heart Fail Clin. 2019;15:77–85. DOI: 10.1016/j.hfc.2018.08.008 30449382

[24] Vogel B, et al. The Lancet women and cardiovascular disease Commission: reducing the global burden by 2030. Lancet. 2021;397:2385–2438. DOI: 10.1016/S0140-6736(21)00684-X 34010613

