# Supplementary material for: Global, Regional, and National Burden of Non-Rheumatic Valvular Heart Diseases in Women: A Systematic Analysis of Global Burden of Disease 1990–2021
Source: Glob Heart. 2025 Mar 26;20(1):33. doi: 10.5334/gh.1422 (PMC11951974; doi:10.5334/gh.1422)
Supplement: Supplementary Materials. — Review search terms. [file gh-20-1-1422-s1.pdf]

Supplementary Materials S1. Mortality data for women with NRVHD from 1990 to 2021.

**Location**	**1990**	
	**Death**	**ASDR**
**Global**	48449.7 (43197.2-52543.3)	2.7 (2.3-2.9)
**SDI**		
Low SDI	1517.7 (845.5-2220.2)	1.6 (0.9-2.4)
Low-middle SDI	3072.6 (2069.9-4367.8)	1.2 (0.8-1.7)
Middle SDI	3630.6 (3009-4463.9)	0.8 (0.6-1)
High-middle SDI	7065.1 (6393.2-7629.3)	1.4 (1.3-1.5)
High SDI	33119.2 (28838.4-35349.7)	4.7 (4.1-5)
**GBD Region**		
Andean Latin America	97.1 (76.6-117.2)	0.9 (0.7-1.1)
Australasia	585.7 (512.3-634.9)	4.2 (3.7-4.6)
Caribbean	165 (141-191.9)	1.2 (1.1-1.4)
Central Asia	40.8 (34.6-48.9)	0.1 (0.1-0.2)
Central Europe	1200.7 (1096.2-1405.9)	1.4 (1.3-1.7)
Central Latin America	530.6 (506.2-550.8)	1.3 (1.2-1.4)
Central Sub-Saharan Africa	194.1 (106.5-286.3)	2.2 (1.2-3.3)
East Asia	884.8 (586.1-1164.7)	0.3 (0.2-0.3)
Eastern Europe	293 (276.3-305.4)	0.2 (0.2-0.2)
Eastern Sub-Saharan Africa	589.8 (326.3-893.1)	1.8 (1-2.9)
High-income Asia Pacific	5531.8 (4784.4-5946.8)	5.2 (4.4-5.6)
High-income North America	10334.5 (8729.6-11156.4)	4.3 (3.7-4.7)
North Africa and Middle East	2029.9 (1343.6-2698.8)	2.5 (1.6-3.3)
Oceania	16.8 (9.9-28.6)	1.3 (0.9-2)
South Asia	2498.3 (1462-3750.6)	1.1 (0.7-1.7)
Southeast Asia	342.9 (225.4-853.7)	0.3 (0.2-0.8)
Southern Latin America	1050.2 (915-1188.3)	4.3 (3.7-4.9)
Southern Sub-Saharan Africa	213.1 (129.9-261.6)	1.4 (0.8-1.8)
Tropical Latin America	1166.8 (1098.6-1209.3)	2.5 (2.3-2.6)
Western Europe	19884.4 (17586.5-21184.7)	5.1 (4.5-5.4)
Western Sub-Saharan Africa	799.5 (447.1-1164.7)	2.1 (1.2-3.2)

* ASDR: Age-Standardized Death Rates

2021		1990-2021
**Death**	**ASDR**	**EAPC**
106463.3 (86729.1-118494.4)	2.2 (1.8-2.5)	-0.142 (-0.246--0.037)
3427.3 (2136.8-4722.6)	1.6 (1-2.2)	-0.112 (-0.212--0.012)
8436.8 (6201.5-10728.3)	1.3 (0.9-1.6)	0.236 (0.172-0.3)
9361.4 (7647.5-11734.4)	0.7 (0.6-0.9)	-0.389 (-0.459--0.319)
18313 (14979.1-20290.9)	1.6 (1.3-1.7)	0.895 (0.715-1.076)
66793.8 (50829.1-75506.5)	3.8 (3-4.3)	-0.267 (-0.374--0.16)
212.5 (160.9-260.8)	0.7 (0.5-0.8)	-1.103 (-1.321--0.885)
1249.6 (968.9-1417.1)	3.2 (2.5-3.6)	-0.506 (-0.706--0.305)
372 (316.1-432.5)	1.3 (1.1-1.5)	-0.026 (-0.171-0.12)
261.3 (230.5-293.6)	0.6 (0.6-0.7)	4.509 (3.99-5.031)
5284.9 (4648.7-5789)	3.5 (3.1-3.8)	3.223 (3.003-3.444)
1579.2 (1380.6-1781.2)	1.2 (1-1.3)	-0.92 (-1.34--0.499)
528.1 (320.5-842.2)	2.3 (1.4-3.8)	0.067 (0.014-0.119)
1424.6 (1054.8-2026.8)	0.1 (0.1-0.2)	-1.96 (-2.323--1.596)
1715.8 (1533.1-1865.1)	0.7 (0.7-0.8)	4.827 (4.465-5.19)
1156 (764.5-1746.6)	1.5 (1-2.4)	-0.657 (-0.74--0.575)
16331.1 (11060-19459.3)	3 (2.1-3.6)	-1.555 (-1.935--1.174)
18347.8 (14274.5-20435.2)	3.9 (3.1-4.3)	0.349 (0.003-0.696)
3983.4 (2892.3-5294.7)	2 (1.4-2.6)	-0.638 (-0.701--0.574)
36.7 (22.5-64.4)	1 (0.7-1.8)	-0.864 (-0.967--0.761)
8606.6 (5805.3-11003.4)	1.3 (0.9-1.7)	0.497 (0.426-0.568)
1011.2 (696.3-2252)	0.4 (0.2-0.8)	0.175 (0.038-0.312)
1761.8 (1494.7-1957.6)	3.1 (2.6-3.4)	-0.538 (-0.679--0.396)
429.4 (288.1-556.9)	1.5 (1-2)	0.206 (0.035-0.378)
2976.9 (2586.1-3219.6)	2.1 (1.8-2.3)	-0.769 (-0.89--0.647)
37842 (29554.3-42546.3)	4.6 (3.7-5.2)	-0.087 (-0.152--0.022)
1352.5 (780.4-2068.4)	1.6 (1-2.6)	-0.754 (-0.891--0.616)

Supplementary Materials S2. YLDs data for women with NRVHD from 1990 to 2021.

**Location**	**1990**		**2021**
	**YLDS**	**ASYLDSR**	**YLDS**
**Global**	73185.6 (41893.7-127257.7)	3.8 (2.2-6.5)	167545.5 (96701.5-287[illegible]
**SDI**			
Low SDI	439.6 (231.9-774.2)	0.5 (0.3-0.9)	1171.3 (630-2084.3)
Low-middle SDI	2168.9 (1161.4-3779.7)	1 (0.5-1.6)	6738.1 (3724.2-11701.9[illegible]
Middle SDI	5719 (3097.4-9656.8)	1.4 (0.8-2.3)	19979.9 (11214.2-3423[illegible]
High-middle SDI	20027.8 (11137.9-34707.5)	3.8 (2.1-6.5)	46022.2 (26453.5-7912[illegible]
High SDI	44740.9 (25722.4-77634.6)	6.2 (3.5-10.7)	93436.9 (53476.1-1581[illegible]
**GBD Region**			
Andean Latin America	91.5 (48.4-158.5)	1 (0.5-1.7)	455.6 (247.5-783.8)
Australasia	593.5 (336.1-1033.4)	4.1 (2.4-7.2)	1572.6 (894.5-2617.2)
Caribbean	145.4 (79.5-249.8)	1.1 (0.6-2)	448.7 (254.7-772.9)
Central Asia	1101.9 (601.9-1903.2)	4.1 (2.3-7.1)	2260 (1284.8-3767.3)
Central Europe	4012.3 (2164.4-6999.7)	4.7 (2.6-8.1)	9293.1 (5275.3-15671.4[illegible]
Central Latin America	545.8 (289.2-964.5)	1.5 (0.8-2.6)	2634.1 (1467.3-4614.2)
Central Sub-Saharan Africa	31.2 (15.8-58.1)	0.4 (0.2-0.7)	83.6 (45.4-154.5)
East Asia	5405 (3001.1-9041.3)	1.6 (0.9-2.6)	17404.5 (9731.2-29942.[illegible]
Eastern Europe	6889.5 (3834.8-12127.4)	3.7 (2.1-6.5)	11948.4 (6664.5-20805.[illegible]
Eastern Sub-Saharan Africa	7546.1 (4215.6-13252.1)	6.6 (3.7-11.6)	23887.1 (13526.7-4067[illegible]
High-income Asia Pacific	20861.2 (11930.9-36382.7)	8.7 (4.9-15.3)	37542 (21931.8-63059.[illegible]
High-income North America	994.2 (529-1724.4)	1.5 (0.8-2.5)	2900.3 (1638.7-4955.4)
North Africa and Middle East	994.2 (529-1724.4)	1.5 (0.8-2.5)	2900.3 (1638.7-4955.4)
Oceania	9.7 (5.3-17.8)	1.1 (0.6-1.9)	27.9 (15.2-47.9)
South Asia	1557.8 (837-2711.2)	0.8 (0.4-1.3)	5582.3 (3077.6-9789.9)
Southeast Asia	1176.2 (653.2-1997.1)	1.1 (0.6-1.9)	3948.2 (2258.4-6603.8)
Southern Latin America	841.4 (474.6-1391.5)	3.3 (1.9-5.4)	2671.6 (1558.7-4609)
Southern Sub-Saharan Africa	56.8 (29.5-103.2)	0.4 (0.2-0.7)	128.3 (69-235.4)
Tropical Latin America	691 (368.1-1198)	1.7 (0.9-2.9)	2992.9 (1615.7-5262.2)
Western Europe	20431 (11832.6-34962.2)	5.1 (2.9-8.7)	41260.2 (23341.6-6931[illegible]
Western Sub-Saharan Africa	99.6 (52.9-179.2)	0.3 (0.1-0.5)	247.4 (132-438.7)

* ASYLDSR: Age-Standardized YLDS Rates

	1990-2021
**ASYLDSR**	**EAPC**
3.6 (2.1-6.1)	-0.038 (-0.099-0.023)
0.6 (0.3-1)	0.31 (0.249-0.372)
1.1 (0.6-1.8)	0.348 (0.288-0.407)
1.5 (0.9-2.6)	0.54 (0.481-0.598)
4 (2.3-6.8)	0.392 (0.293-0.491)
6.5 (3.6-11.2)	0.312 (0.256-0.368)
1.5 (0.8-2.6)	1.34 (1.287-1.393)
4.5 (2.6-7.7)	0.381 (0.308-0.454)
1.5 (0.8-2.6)	0.854 (0.801-0.907)
5.8 (3.3-9.6)	1.451 (1.22-1.682)
6.2 (3.5-10.6)	1.318 (1.038-1.599)
2 (1.1-3.5)	1.276 (1.12-1.433)
0.4 (0.2-0.7)	0.059 (-0.052-0.171)
1.6 (0.9-2.7)	0.178 (0.091-0.266)
4.8 (2.7-8.4)	1.096 (0.976-1.217)
6.5 (3.6-11.5)	0.02 (-0.078-0.118)
8.9 (5.1-15.2)	0.286 (0.205-0.367)
1.5 (0.9-2.6)	0.352 (0.256-0.447)
1.5 (0.9-2.6)	0.161 (0.132-0.19)
1.2 (0.7-2)	0.354 (0.295-0.412)
0.9 (0.5-1.5)	0.493 (0.425-0.561)
1.3 (0.8-2.2)	1.095 (0.851-1.34)
4.8 (2.8-8.2)	0.031 (-0.033-0.095)
0.4 (0.2-0.8)	0.145 (0.114-0.175)
2.1 (1.1-3.7)	0.764 (0.646-0.882)
6.1 (3.4-10.4)	0.776 (0.645-0.907)
0.3 (0.2-0.5)	0.372 (0.346-0.398)

Supplementary Materials S3. YLLs data for women with NRVHD from 1990 to 2021.

**Location**	**1990**		**2021**
	**YLLS**	**ASYLLSR**	**YLLS**
**Global**	859747.6 (762759.4-962895.3)	42.5 (37.9-47.1)	1513846.2 (1299392.2-169703[illegible]
**SDI**			
Low SDI	44970.4 (22952.9-67765.7)	35.3 (19.6-51.5)	94074.7 (60485.9-126963.1)
Low-middle SDI	87701.8 (56231.5-126634.3)	26 (17.6-36.6)	204456 (151676.8-263421.8)
Middle SDI	103864.9 (85854-127365.7)	17.6 (14.5-21.5)	207222.5 (174428.4-259915.7)
High-middle SDI	134303.9 (119642.6-147777.5)	25.1 (22.3-27.6)	255798.1 (217310.7-282435)
High SDI	488007.2 (440108.7-513711.1)	72 (65.6-75.6)	750249.9 (599142.1-833239.2)
**GBD Region**			
Andean Latin America	3003.3 (2305.9-3640.8)	23.1 (17.9-27.9)	5354.5 (4021.9-6703.2)
Australasia	8755 (7858.3-9361.6)	64.4 (58.1-68.7)	14391.8 (11530.5-16075.4)
Caribbean	4667.7 (3817-5670.5)	31.7 (26.4-37.9)	8772.6 (7195.9-10557.2)
Central Asia	1123.8 (965.1-1274.5)	3.8 (3.2-4.3)	5762 (5092.2-6420.1)
Central Europe	24359.1 (22381.3-28107.3)	28.9 (26.6-33.3)	78254.4 (69744-85471.6)
Central Latin America	14359.9 (13889.9-14856.8)	29.3 (28.2-30.4)	35985.7 (31666-40759.3)
Central Sub-Saharan Africa	5545.9 (2953.6-8181.5)	44.9 (25.1-66.5)	13807.2 (8310-21805.4)
East Asia	23594.5 (15037.4-31871.5)	5.2 (3.4-6.9)	26183.1 (19744.5-39235.1)
Eastern Europe	5994.8 (5728.7-6235.6)	3.6 (3.5-3.8)	31480.4 (28375.7-34441)
Eastern Sub-Saharan Africa	18975.7 (9681.9-28695.8)	39.9 (21.9-60.4)	33666.9 (22756-48781)
High-income Asia Pacific	85411.9 (76633.1-90500.4)	77.7 (69.1-82.6)	159636 (111096.7-188455.9)
High-income North America	152333 (134794.3-161388.5)	69.7 (62.7-73.4)	218411.8 (179676.7-238009.3)
North Africa and Middle East	64091.3 (41174.9-87643.3)	63.6 (42-85.5)	109038.9 (80980.1-145565.2)
Oceania	707.3 (363.7-1344.7)	32.8 (19.4-55.7)	1590.9 (903.4-2825.1)
South Asia	67577.4 (38467.8-107821.4)	23.3 (13.5-35.2)	194797.6 (131827.4-254202.1)
Southeast Asia	9005.2 (5852.5-22923.4)	6.1 (4.1-15.5)	20499.5 (14405.7-47679)
Southern Latin America	17614.5 (15736.9-19729.1)	70 (62.5-78.4)	24735.5 (21717.5-27171.6)
Southern Sub-Saharan Africa	6688.9 (4192.8-7990.8)	34.3 (21.3-41.2)	10455.7 (7451.5-13808.9)
Tropical Latin America	33786.8 (32635.6-34654.8)	61.1 (58.3-62.9)	62311.6 (56332.3-66139.7)
Western Europe	292036.6 (265653-307847.4)	80.1 (73.7-84.1)	423512.4 (341094.9-471382.3)

Western Sub-Saharan Africa	20115 (11122.5-28757.3)	42.5 (23.8-61.9)	35197.6 (19839.6-52582.3)

* ASYLLSR: Age-Standardized YLLS Rates

	1990-2021
**ASYLLSR**	**EAPC**
32.8 (28.2-36.8)	-0.638 (-0.71--0.566)
32.1 (19.8-44.3)	-0.344 (-0.423--0.265)
26.6 (19.9-34.1)	0.056 (0.009-0.103)
15.2 (12.7-19.1)	-0.714 (-0.803--0.625)
23.3 (20-25.8)	0.144 (0.008-0.281)
50.3 (41.8-54.9)	-0.943 (-1.043--0.843)
16.7 (12.6-21)	-1.37 (-1.596--1.143)
41.4 (34.1-45.7)	-1.045 (-1.25--0.84)
31.8 (25.8-38.5)	-0.033 (-0.165-0.099)
12.9 (11.4-14.4)	3.758 (3.312-4.206)
57.4 (51.4-62.7)	2.568 (2.364-2.772)
26.5 (23.3-29.9)	-0.996 (-1.407--0.582)
44.9 (27.1-71.5)	-0.062 (-0.109--0.015)
2.5 (1.9-3.8)	-2.336 (-2.648--2.023)
15.4 (13.9-16.9)	4.731 (4.435-5.029)
31 (20.5-46.6)	-0.907 (-1.002--0.812)
34.9 (26.1-40.2)	-2.57 (-2.978--2.161)
52.3 (44.5-56.3)	-0.418 (-0.732--0.104)
44.9 (33.2-59.7)	-0.949 (-1.006--0.892)
28.7 (17.4-49.9)	-0.49 (-0.579--0.402)
26 (17.6-33.7)	0.327 (0.28-0.375)
6.3 (4.4-14.3)	-0.143 (-0.268--0.018)
46.6 (41.4-50.8)	-0.823 (-0.953--0.692)
30.8 (21.4-40.4)	-0.469 (-0.698--0.239)
44.6 (40.4-47.3)	-1.409 (-1.58--1.238)
60.4 (50.2-66.1)	-0.759 (-0.823--0.694)

31.1 (17.9-47.6)	-0.918 (-1.064--0.772)

Supplementary Materials S4. Epidemiological data of female NRVHD from 1990 to 2021 across 204 c

	1990			
**Location**	**Incidence**	**Prevalence**	**Deaths**	**YLDs**
**Afghanistan**	239.6 (207.0 – 281.0	2237.2 (1881.6 – 2706.9)	61.8 (27.9 – 118.0)	27.8 (14.5 – 52.3)
**Albania**	225.7 (193.1 – 266.1	2928.3 (2467.7 – 3470.5)	14.1 (9.3 – 21.8)	41.5 (22.7 – 72.4)
**Algeria**	640.9 (560.6 – 730.9	6674.0 (5732.1 – 7740.7)	115.7 (72.0 – 165.7)	84.5 (43.1 – 146.8)
**American Samoa**	0.8 (0.7 – 0.9)	8.2 (6.9 – 9.7)	0.1 (0.1 – 0.2)	0.1 (0.1 – 0.2)
**Andorra**	6.2 (5.3 – 7.2)	66.9 (57.1 – 78.4)	0.9 (0.6 – 1.3)	0.9 (0.5 – 1.5)
**Angola**	60.3 (53.1 – 68.7)	461.9 (388.2 – 550.9)	34.5 (18.4 – 54.8)	5.1 (2.6 – 9.4)
**Antigua and Barbuda**	1.6 (1.4 – 1.8)	23.8 (19.9 – 27.8)	0.3 (0.3 – 0.4)	0.4 (0.2 – 0.7)
**Argentina**	3566.4 (3099.5 – 409	37372.1 (31880.2 – 43277.1)	844.5 (726.5 – 973.5	604.9 (333.2 – 994.8
**Armenia**	251.6 (207.6 – 306.3	3318.1 (2734.3 – 3998.2)	4.1 (3.3 – 5.0)	46.5 (24.7 – 81.5)
**Australia**	2830.2 (2427.1 – 324	31016.7 (26289.2 – 35579.5)	460.4 (402.7 – 502.9	469.2 (262.1 – 809.9
**Austria**	3440.6 (3003.4 – 411	50581.7 (44209.1 – 61273.2)	319.5 (285.2 – 346.2	775.1 (427.7 – 1365.
**Azerbaijan**	553.1 (455.8 – 672.8	7474.5 (6189.5 – 9036.4)	2.6 (1.8 – 3.7)	111.9 (62.8 – 200.4)
**Bahamas**	5.8 (4.9 – 6.7)	73.1 (61.5 – 87.5)	0.9 (0.8 – 1.0)	1.0 (0.5 – 1.7)
**Bahrain**	8.8 (7.6 – 9.9)	83.6 (70.7 – 96.3)	1.9 (1.4 – 2.6)	0.9 (0.4 – 1.6)
**Bangladesh**	946.1 (802.9 – 1091.	9098.5 (7443.5 – 10791.2)	227.6 (115.7 – 372.1	126.4 (67.3 – 220.4)
**Barbados**	9.9 (8.4 – 11.5)	153.8 (129.0 – 181.4)	1.6 (1.4 – 1.9)	2.5 (1.3 – 4.2)
**Belarus**	1514.8 (1258.6 – 181	21940.4 (18257.5 – 25987.7)	2.8 (2.3 – 3.3)	331.8 (184.8 – 583.0
**Belgium**	2101.3 (1800.0 – 244	23291.2 (19418.4 – 27641.9)	850.6 (709.7 – 950.6	368.7 (206.3 – 639.6
**Belize**	2.3 (2.0 – 2.7)	31.0 (25.9 – 36.1)	0.3 (0.3 – 0.4)	0.5 (0.2 – 0.8)
**Benin**	23.0 (20.1 – 26.1)	200.3 (168.0 – 239.0)	12.1 (6.4 – 19.0)	2.4 (1.2 – 4.2)
**Bermuda**	5.2 (3.7 – 6.4)	68.3 (50.4 – 85.7)	1.5 (1.3 – 1.7)	0.9 (0.5 – 1.7)
**Bhutan**	5.5 (4.7 – 6.4)	51.9 (43.0 – 62.0)	1.1 (0.5 – 1.9)	0.7 (0.3 – 1.3)
**Bolivia (Plurinational State of)**	98.4 (85.8 – 113.1)	1124.8 (960.7 – 1329.2)	22.6 (11.4 – 34.2)	14.1 (7.3 – 26.3)
**Bosnia and Herzegovina**	573.0 (491.3 – 670.8	6816.5 (5760.1 – 7948.7)	45.6 (29.5 – 70.8)	88.4 (47.6 – 152.3)
**Botswana**	9.0 (7.8 – 10.2)	87.4 (72.1 – 103.5)	3.9 (2.4 – 5.7)	0.9 (0.5 – 1.8)
**Brazil**	4995.0 (4420.3 – 568	56259.8 (49645.1 – 64300.1)	1142.8 (1078.4 – 118	674.0 (357.7 – 1168.
**Brunei Darussalam**	17.2 (14.7 – 20.3)	193.8 (165.0 – 228.4)	1.7 (1.2 – 2.5)	2.8 (1.5 – 4.9)
**Bulgaria**	1454.8 (1203.8 – 174	20001.1 (16749.2 – 23596.7)	24.6 (21.2 – 28.2)	263.8 (145.1 – 470.4
**Burkina Faso**	46.3 (39.8 – 52.6)	401.6 (327.1 – 479.0)	30.3 (13.6 – 47.4)	4.5 (2.3 – 8.2)
**Burundi**	42.3 (37.7 – 47.3)	323.6 (271.9 – 382.4)	25.9 (13.3 – 44.5)	3.9 (2.0 – 7.3)
**Cabo Verde**	2.6 (2.3 – 2.9)	28.0 (23.5 – 33.1)	1.9 (0.9 – 3.9)	0.4 (0.2 – 0.7)
**Cambodia**	121.1 (99.7 – 147.5)	1244.7 (1005.2 – 1542.3)	4.2 (1.6 – 15.8)	17.6 (8.5 – 30.8)
**Cameroon**	58.6 (51.4 – 66.8)	512.2 (421.7 – 606.5)	43.6 (23.3 – 67.9)	5.6 (2.8 – 9.9)
**Canada**	6977.7 (5958.3 – 817	88845.5 (76472.0 – 104923.2	616.7 (544.5 – 669.9	1242.1 (693.6 – 213
**Central African Republic**	18.7 (16.4 – 21.3)	133.1 (109.6 – 160.0)	11.4 (6.0 – 18.4)	1.5 (0.7 – 2.8)
**Chad**	28.9 (25.3 – 32.8)	244.6 (202.1 – 292.1)	21.3 (9.4 – 35.7)	3.0 (1.6 – 5.5)
**Chile**	867.6 (741.0 – 996.7	10125.7 (8637.3 – 11743.3)	92.1 (84.7 – 98.7)	152.4 (85.9 – 263.7)
**China**	31854.9 (29802.0 – 3	342956.7 (319757.3 – 36924	721.5 (412.5 – 992.4	5146.5 (2841.9 – 859
**Colombia**	921.4 (800.9 – 1062.	10718.8 (9090.4 – 12525.3)	206.1 (194.1 – 217.0	136.5 (71.8 – 237.3)
**Comoros**	3.2 (2.8 – 3.6)	26.1 (21.8 – 31.6)	1.8 (0.9 – 2.8)	0.3 (0.2 – 0.6)
**Congo**	20.4 (18.0 – 22.9)	160.6 (133.6 – 191.1)	14.0 (7.6 – 21.4)	1.9 (1.0 – 3.6)
**Cook Islands**	0.3 (0.3 – 0.4)	4.1 (3.4 – 4.8)	0.1 (0.1 – 0.1)	0.1 (0.0 – 0.1)
**Costa Rica**	83.8 (72.0 – 96.7)	1065.9 (902.9 – 1246.7)	11.0 (9.8 – 12.2)	14.4 (7.7 – 25.4)
**Croatia**	1041.5 (883.1 – 1215	14056.2 (11839.7 – 16341.8)	79.3 (70.6 – 89.6)	180.4 (94.3 – 309.4)
**Cuba**	308.6 (264.9 – 352.6	4309.3 (3639.8 – 4996.2)	57.8 (51.1 – 61.9)	55.8 (29.7 – 99.5)
**Cyprus**	156.1 (123.5 – 191.2	1273.5 (1026.8 – 1577.6)	53.6 (33.2 – 81.3)	19.1 (9.8 – 33.7)

**Czechia**	1845.0 (1536.7 – 216	29313.0 (24573.1 – 33849.9)	68.1 (62.9 – 73.6)	415.3 (233.2 – 709.7
**Côte d'Ivoire**	45.8 (39.4 – 52.8)	387.1 (314.4 – 466.3)	25.3 (13.0 – 40.3)	3.7 (1.9 – 6.9)
**Democratic People's Republic of K**	703.9 (571.2 – 864.6	7375.4 (5972.5 – 9069.2)	23.6 (12.0 – 67.8)	115.6 (62.0 – 206.5)
**Democratic Republic of the Congo**	245.4 (214.6 – 277.2	1903.6 (1573.0 – 2292.2)	125.0 (67.8 – 192.7)	21.0 (10.6 – 39.3)
**Denmark**	1048.9 (896.7 – 1239	13295.5 (11390.3 – 15504.0)	199.6 (175.5 – 221.4	216.6 (121.1 – 369.9
**Djibouti**	2.1 (1.8 – 2.3)	16.5 (13.7 – 19.7)	1.1 (0.6 – 1.6)	0.2 (0.1 – 0.3)
**Dominica**	1.6 (1.3 – 1.9)	22.8 (18.8 – 27.1)	0.1 (0.1 – 0.2)	0.3 (0.2 – 0.6)
**Dominican Republic**	98.5 (84.1 – 114.5)	1170.0 (971.4 – 1379.3)	15.8 (10.4 – 19.9)	14.4 (7.3 – 25.2)
**Ecuador**	168.4 (144.1 – 195.5	2089.9 (1747.9 – 2469.6)	40.2 (35.8 – 43.7)	27.4 (14.2 – 47.4)
**Egypt**	1253.2 (1104.4 – 143	11730.5 (10039.2 – 13731.7)	316.2 (214.2 – 441.1	133.8 (67.4 – 239.6)
**El Salvador**	86.1 (72.2 – 103.1)	1110.6 (929.4 – 1332.2)	3.5 (2.4 – 4.7)	15.7 (8.7 – 28.4)
**Equatorial Guinea**	3.2 (2.8 – 3.6)	25.1 (21.1 – 29.9)	2.0 (1.0 – 3.2)	0.3 (0.1 – 0.5)
**Eritrea**	19.4 (16.9 – 22.0)	142.2 (118.4 – 170.3)	11.3 (5.5 – 18.3)	1.4 (0.7 – 2.7)
**Estonia**	295.1 (246.5 – 346.3	4357.3 (3681.7 – 5055.0)	11.9 (10.5 – 13.6)	67.6 (36.5 – 113.2)
**Eswatini**	5.0 (4.4 – 5.6)	46.2 (38.4 – 54.5)	2.2 (1.4 – 3.1)	0.5 (0.3 – 0.9)
**Ethiopia**	279.7 (253.5 – 306.0	2033.8 (1804.2 – 2278.3)	139.6 (63.5 – 236.3)	21.5 (10.8 – 39.8)
**Fiji**	9.9 (8.3 – 11.9)	101.1 (82.9 – 122.8)	0.6 (0.4 – 0.8)	1.4 (0.7 – 2.5)
**Finland**	1888.5 (1544.0 – 268	23848.6 (19333.1 – 33778.5)	270.3 (221.9 – 321.2	386.0 (212.6 – 687.1
**France**	12964.2 (10993.2 – 1	153360.4 (127754.9 – 17992	3828.7 (3344.0 – 417	2565.1 (1481.1 – 427
**Gabon**	11.8 (10.5 – 13.2)	102.2 (85.9 – 121.8)	7.2 (4.3 – 10.6)	1.3 (0.7 – 2.5)
**Gambia**	3.6 (3.1 – 4.2)	30.8 (25.4 – 36.8)	2.7 (1.2 – 4.4)	0.4 (0.2 – 0.6)
**Georgia**	920.7 (757.0 – 1121.	13196.6 (10826.0 – 16064.9)	5.7 (4.7 – 6.6)	197.9 (110.9 – 344.4
**Germany**	22324.4 (19605.4 – 2	274267.6 (234883.0 – 31999	5919.0 (5135.9 – 654	4408.3 (2529.1 – 735
**Ghana**	81.1 (70.9 – 92.1)	707.5 (586.9 – 848.7)	72.4 (41.7 – 101.9)	7.8 (4.2 – 14.0)
**Greece**	1664.4 (1455.9 – 187	18859.3 (16158.9 – 21572.7)	337.6 (296.4 – 363.9	262.4 (142.5 – 467.0
**Greenland**	8.1 (6.9 – 9.4)	82.6 (69.8 – 95.9)	0.9 (0.4 – 1.3)	1.2 (0.7 – 2.0)
**Grenada**	1.8 (1.5 – 2.1)	26.7 (22.6 – 31.3)	0.3 (0.3 – 0.4)	0.4 (0.2 – 0.8)
**Guam**	3.3 (2.7 – 3.9)	33.0 (27.6 – 39.4)	0.9 (0.6 – 1.2)	0.4 (0.2 – 0.8)
**Guatemala**	99.5 (84.1 – 115.6)	1084.2 (905.1 – 1283.2)	8.4 (6.3 – 10.5)	13.3 (6.8 – 24.1)
**Guinea**	38.2 (33.7 – 43.0)	325.7 (269.2 – 388.5)	27.5 (12.9 – 44.2)	3.9 (2.1 – 7.2)
**Guinea-Bissau**	4.8 (4.2 – 5.4)	38.3 (31.4 – 46.2)	4.1 (2.1 – 6.4)	0.4 (0.2 – 0.7)
**Guyana**	14.7 (12.9 – 16.6)	161.8 (137.7 – 187.2)	4.1 (3.4 – 4.8)	2.0 (1.1 – 3.5)
**Haiti**	91.3 (79.2 – 104.4)	931.2 (772.2 – 1092.0)	32.6 (16.3 – 54.5)	11.0 (5.5 – 20.4)
**Honduras**	59.7 (50.7 – 70.2)	686.4 (572.3 – 820.4)	10.9 (6.2 – 14.9)	9.1 (4.9 – 16.1)
**Hungary**	2901.2 (2458.6 – 335	43094.0 (36965.9 – 49609.5)	286.6 (260.1 – 311.0	599.4 (324.8 – 1022.
**Iceland**	42.7 (36.5 – 50.7)	520.3 (433.6 – 631.9)	8.5 (7.1 – 9.4)	7.6 (4.2 – 13.3)
**India**	10398.6 (9569.4 – 11	95401.6 (86475.5 – 104801.2	1968.7 (1166.6 – 296	1238.1 (666.5 – 2158
**Indonesia**	2662.2 (2458.0 – 288	26853.7 (24639.6 – 29398.8)	100.8 (41.8 – 369.2)	382.9 (211.6 – 671.9
**Iran (Islamic Republic of)**	1015.4 (941.3 – 1094	10223.9 (9319.7 – 11215.9)	193.8 (139.2 – 249.4	117.3 (62.7 – 203.9)
**Iraq**	257.0 (222.0 – 300.3	2981.8 (2532.5 – 3522.6)	53.3 (37.6 – 75.3)	42.3 (22.8 – 75.6)
**Ireland**	500.8 (439.7 – 576.6	5931.5 (5072.1 – 6850.4)	81.9 (73.5 – 88.6)	87.8 (49.3 – 151.9)
**Israel**	578.3 (508.6 – 644.5	5672.2 (4902.4 – 6410.0)	156.9 (138.7 – 173.1	86.1 (48.3 – 152.6)
**Italy**	25941.7 (23857.6 – 2	367497.1 (342059.7 – 39612	1328.9 (1188.0 – 140	5230.8 (2958.5 – 911
**Jamaica**	45.2 (38.6 – 52.7)	653.3 (544.5 – 763.8)	4.0 (3.5 – 4.5)	9.9 (5.6 – 17.7)
**Japan**	39119.9 (35047.1 – 4	488682.0 (439135.3 – 54771	5310.4 (4556.8 – 571	6801.5 (3773.1 – 119
**Jordan**	48.5 (41.5 – 55.9)	510.3 (424.3 – 595.8)	9.3 (5.8 – 12.5)	5.7 (3.0 – 10.1)
**Kazakhstan**	1540.4 (1270.4 – 186	21183.9 (17426.8 – 25449.2)	11.7 (9.4 – 14.6)	309.9 (171.2 – 531.7
**Kenya**	120.1 (108.0 – 131.7	1012.0 (899.0 – 1146.2)	49.7 (30.6 – 70.2)	13.2 (7.3 – 23.5)
**Kiribati**	1.2 (1.0 – 1.5)	12.6 (10.4 – 15.3)	0.8 (0.6 – 1.2)	0.2 (0.1 – 0.3)
**Kuwait**	21.8 (18.8 – 25.3)	239.2 (204.5 – 281.3)	2.6 (2.4 – 2.9)	2.3 (1.2 – 4.1)

**Kyrgyzstan**	333.2 (275.9 – 407.5	4657.7 (3865.1 – 5720.1)	1.5 (1.3 – 1.7)	70.6 (37.9 – 126.0)
**Lao People's Democratic Republic**	55.9 (46.0 – 68.4)	564.6 (459.6 – 690.3)	2.3 (0.8 – 9.2)	8.1 (4.0 – 14.4)
**Latvia**	484.8 (405.0 – 570.3	7146.2 (6056.2 – 8289.3)	10.7 (9.5 – 12.2)	110.0 (59.6 – 183.4)
**Lebanon**	99.1 (86.4 – 113.3)	1108.0 (936.5 – 1286.3)	52.7 (30.0 – 83.8)	14.4 (7.9 – 25.9)
**Lesotho**	13.3 (11.7 – 15.0)	126.0 (103.0 – 151.2)	5.7 (3.4 – 8.4)	1.6 (0.8 – 3.0)
**Liberia**	11.7 (10.2 – 13.4)	100.5 (82.8 – 118.8)	8.9 (4.5 – 13.3)	1.2 (0.6 – 2.2)
**Libya**	74.8 (65.3 – 86.4)	847.8 (721.9 – 985.8)	14.9 (9.5 – 22.5)	11.8 (6.7 – 20.8)
**Lithuania**	560.7 (468.6 – 671.9	8214.1 (6926.6 – 9638.2)	7.4 (6.5 – 8.4)	124.3 (70.3 – 216.4)
**Luxembourg**	134.0 (117.8 – 150.5	1624.3 (1396.9 – 1847.6)	27.0 (24.4 – 29.3)	26.0 (15.1 – 44.2)
**Madagascar**	87.6 (78.2 – 97.5)	679.8 (574.4 – 796.1)	66.2 (35.7 – 99.4)	7.7 (4.1 – 14.1)
**Malawi**	58.1 (51.0 – 66.2)	457.1 (382.6 – 543.5)	23.1 (12.1 – 36.3)	5.5 (2.9 – 10.4)
**Malaysia**	302.9 (256.6 – 359.4	3408.7 (2837.2 – 4088.4)	31.4 (17.8 – 40.0)	53.3 (29.0 – 90.6)
**Maldives**	3.2 (2.6 – 3.8)	27.6 (22.3 – 33.5)	0.3 (0.2 – 0.7)	0.3 (0.2 – 0.6)
**Mali**	37.4 (32.1 – 43.0)	314.7 (256.9 – 381.2)	31.9 (15.5 – 51.9)	3.3 (1.7 – 6.2)
**Malta**	35.9 (31.5 – 40.5)	403.3 (348.9 – 464.0)	6.5 (5.7 – 7.1)	5.9 (3.2 – 10.5)
**Marshall Islands**	0.5 (0.4 – 0.6)	5.4 (4.5 – 6.4)	0.2 (0.1 – 0.3)	0.1 (0.0 – 0.1)
**Mauritania**	12.1 (10.7 – 13.6)	110.4 (91.3 – 131.3)	11.8 (6.8 – 17.3)	1.3 (0.7 – 2.5)
**Mauritius**	27.2 (22.5 – 32.5)	313.6 (259.4 – 379.0)	5.8 (5.3 – 6.4)	4.7 (2.5 – 8.2)
**Mexico**	1772.5 (1521.3 – 204[illegible]	21203.1 (18151.5 – 24698.2)	216.5 (208.6 – 222.2	272.1 (142.7 – 485.6
**Micronesia (Federated States of)**	1.6 (1.4 – 1.9)	17.9 (14.9 – 21.6)	0.5 (0.3 – 0.9)	0.3 (0.1 – 0.5)
**Monaco**	6.4 (5.3 – 7.7)	92.7 (77.6 – 108.4)	0.9 (0.6 – 1.4)	1.5 (0.8 – 2.6)
**Mongolia**	112.6 (91.7 – 136.3)	1513.7 (1242.9 – 1792.1)	2.6 (1.4 – 5.0)	22.0 (11.3 – 38.4)
**Montenegro**	72.5 (60.7 – 85.4)	1026.4 (869.9 – 1211.1)	3.2 (2.2 – 4.5)	13.3 (7.1 – 23.2)
**Morocco**	549.4 (481.3 – 634.2	5821.8 (4993.3 – 6854.3)	139.1 (86.8 – 208.5)	77.9 (40.6 – 138.4)
**Mozambique**	88.0 (77.2 – 99.8)	674.7 (558.7 – 799.9)	45.2 (22.9 – 73.9)	8.1 (4.2 – 15.2)
**Myanmar**	622.2 (503.5 – 757.2	6361.6 (5115.7 – 7811.2)	26.1 (11.4 – 89.6)	92.2 (46.4 – 164.4)
**Namibia**	10.7 (9.3 – 12.0)	105.1 (86.2 – 123.6)	4.3 (2.8 – 5.9)	1.2 (0.6 – 2.2)
**Nauru**	0.2 (0.1 – 0.2)	1.4 (1.1 – 1.6)	0.0 (0.0 – 0.1)	0.0 (0.0 – 0.0)
**Nepal**	205.7 (174.4 – 241.0	1882.7 (1543.5 – 2281.5)	41.5 (21.8 – 67.2)	24.5 (12.7 – 45.4)
**Netherlands**	2520.5 (2199.6 – 292[illegible]	27521.7 (23324.0 – 32134.3)	1176.3 (1010.0 – 129[illegible]	398.8 (218.5 – 704.3
**New Zealand**	751.1 (642.2 – 861.9	7980.0 (6952.4 – 9168.3)	125.4 (107.2 – 137.3	124.3 (68.6 – 221.1)
**Nicaragua**	49.5 (42.2 – 57.5)	608.4 (514.1 – 720.9)	2.8 (2.1 – 4.0)	8.0 (4.2 – 13.6)
**Niger**	28.8 (24.9 – 33.1)	230.5 (190.1 – 277.2)	19.4 (8.4 – 34.1)	2.3 (1.2 – 4.2)
**Nigeria**	473.0 (429.5 – 516.0	4187.7 (3724.0 – 4685.9)	435.6 (234.7 – 662.4	52.6 (28.3 – 93.6)
**Niue**	0.1 (0.1 – 0.1)	1.2 (1.0 – 1.4)	0.0 (0.0 – 0.0)	0.0 (0.0 – 0.0)
**North Macedonia**	202.5 (170.4 – 239.7	2594.4 (2194.9 – 3052.8)	8.6 (6.4 – 13.6)	34.7 (19.1 – 60.8)
**Northern Mariana Islands**	0.7 (0.6 – 0.8)	6.7 (5.7 – 7.8)	0.2 (0.1 – 0.3)	0.1 (0.0 – 0.1)
**Norway**	2074.0 (1866.7 – 229[illegible]	26097.9 (23785.7 – 28365.1)	379.0 (326.8 – 408.7	428.8 (245.3 – 731.5
**Oman**	18.5 (15.7 – 21.8)	212.5 (177.0 – 252.5)	2.3 (1.2 – 3.6)	2.6 (1.4 – 4.7)
**Pakistan**	1267.6 (1168.0 – 137[illegible]	11963.6 (10888.4 – 13224.8)	259.5 (154.6 – 423.2	168.2 (92.1 – 294.1)
**Palau**	0.4 (0.3 – 0.4)	4.1 (3.4 – 4.8)	0.1 (0.0 – 0.1)	0.1 (0.0 – 0.1)
**Palestine**	29.6 (25.3 – 34.4)	336.2 (283.3 – 395.3)	3.4 (2.2 – 4.9)	4.1 (2.2 – 7.2)
**Panama**	55.1 (47.7 – 63.8)	699.7 (595.6 – 816.5)	5.2 (4.7 – 5.8)	9.6 (5.0 – 16.9)
**Papua New Guinea**	43.7 (36.0 – 52.6)	404.0 (331.8 – 496.4)	10.3 (4.9 – 20.3)	5.1 (2.6 – 9.6)
**Paraguay**	103.9 (90.4 – 118.3)	1250.8 (1065.4 – 1441.7)	24.0 (17.4 – 34.9)	17.0 (9.3 – 29.9)
**Peru**	303.8 (261.0 – 352.1	3817.5 (3226.8 – 4538.3)	34.3 (23.5 – 46.9)	50.0 (25.7 – 84.3)
**Philippines**	743.0 (683.8 – 809.3	7880.1 (7206.2 – 8626.7)	18.5 (9.5 – 22.8)	112.0 (62.6 – 194.6)
**Poland**	3186.3 (2919.9 – 349[illegible]	48581.6 (45024.0 – 52632.3)	146.4 (138.7 – 151.9	673.3 (368.1 – 1151.[illegible]
**Portugal**	708.8 (623.1 – 807.8	7657.6 (6624.1 – 8748.8)	199.5 (181.6 – 213.3	116.8 (63.4 – 200.5)
**Puerto Rico**	179.6 (154.2 – 206.7	2336.1 (1938.2 – 2726.9)	30.4 (28.0 – 32.8)	33.0 (17.7 – 56.8)

**Qatar**	6.0 (5.2 – 6.9)	53.5 (45.4 – 62.7)	1.1 (0.8 – 1.6)	0.5 (0.3 – 0.9)
**Republic of Korea**	4320.5 (3636.0 – 502	48339.1 (41084.5 – 57011.0)	204.2 (138.5 – 331.1	683.6 (374.1 – 1207.
**Republic of Moldova**	500.7 (417.9 – 604.1	6423.9 (5346.6 – 7654.6)	2.3 (2.1 – 2.6)	89.3 (47.4 – 152.5)
**Romania**	5673.2 (4790.3 – 673	79051.2 (66376.0 – 93745.7)	162.8 (144.3 – 184.2	1089.0 (568.3 – 1898
**Russian Federation**	21517.3 (19421.6 – 2	305379.0 (277507.1 – 33627	227.9 (216.5 – 235.3	4512.4 (2493.8 – 794
**Rwanda**	62.4 (55.7 – 69.7)	468.9 (394.9 – 556.4)	40.1 (23.2 – 62.3)	5.2 (2.6 – 9.6)
**Saint Kitts and Nevis**	1.5 (1.3 – 1.8)	21.1 (17.6 – 24.6)	0.4 (0.3 – 0.5)	0.3 (0.2 – 0.5)
**Saint Lucia**	3.0 (2.6 – 3.5)	39.0 (32.6 – 45.5)	0.6 (0.5 – 0.7)	0.5 (0.3 – 0.9)
**Saint Vincent and the Grenadines**	2.2 (1.9 – 2.5)	28.6 (24.2 – 33.4)	0.4 (0.3 – 0.4)	0.4 (0.2 – 0.7)
**Samoa**	2.8 (2.4 – 3.4)	30.7 (25.6 – 36.8)	0.7 (0.4 – 1.1)	0.5 (0.2 – 0.8)
**San Marino**	4.1 (3.5 – 4.7)	49.3 (41.6 – 57.5)	0.7 (0.5 – 1.0)	0.7 (0.4 – 1.2)
**Sao Tome and Principe**	0.7 (0.6 – 0.8)	6.7 (5.5 – 8.0)	0.5 (0.3 – 0.8)	0.1 (0.0 – 0.2)
**Saudi Arabia**	217.8 (191.6 – 245.7	2329.5 (1999.9 – 2695.4)	47.9 (32.3 – 68.5)	28.6 (15.3 – 49.4)
**Senegal**	33.0 (28.4 – 37.6)	294.4 (241.3 – 353.4)	24.7 (12.4 – 38.0)	3.4 (1.7 – 6.2)
**Serbia**	2114.0 (1814.5 – 241	25069.5 (21189.1 – 28838.8)	246.5 (185.3 – 394.4	319.6 (175.4 – 553.5
**Seychelles**	2.8 (2.4 – 3.3)	36.3 (30.3 – 44.5)	0.4 (0.2 – 0.5)	0.6 (0.3 – 1.1)
**Sierra Leone**	19.6 (17.2 – 22.4)	168.2 (138.9 – 200.6)	14.5 (6.8 – 22.7)	2.1 (1.0 – 3.7)
**Singapore**	346.7 (295.8 – 411.1	4114.6 (3523.5 – 4832.0)	15.6 (14.0 – 16.8)	58.2 (31.3 – 101.9)
**Slovakia**	468.1 (389.4 – 546.7	6936.1 (5777.5 – 8107.0)	32.7 (24.0 – 57.8)	96.4 (53.6 – 176.8)
**Slovenia**	673.5 (578.4 – 779.5	9384.9 (8017.5 – 10911.3)	62.8 (57.2 – 68.4)	133.0 (73.8 – 236.5)
**Solomon Islands**	3.5 (3.0 – 4.2)	32.0 (26.7 – 39.0)	0.4 (0.2 – 0.8)	0.4 (0.2 – 0.7)
**Somalia**	34.7 (30.3 – 39.3)	238.5 (198.7 – 286.0)	18.5 (7.5 – 33.2)	2.5 (1.3 – 4.6)
**South Africa**	392.3 (356.0 – 430.5	3821.7 (3406.3 – 4288.2)	173.0 (104.3 – 212.7	46.1 (24.2 – 83.7)
**South Sudan**	32.1 (28.6 – 36.2)	263.9 (221.0 – 309.1)	16.6 (8.2 – 28.3)	3.4 (1.7 – 6.3)
**Spain**	6547.8 (5715.3 – 737	76749.1 (65800.6 – 87932.6)	1766.3 (1539.7 – 193	1124.5 (619.0 – 1954
**Sri Lanka**	491.6 (409.7 – 593.7	5067.9 (4194.9 – 6047.1)	38.3 (19.1 – 48.2)	73.8 (40.1 – 131.1)
**Sudan**	310.8 (274.0 – 352.3	3208.3 (2743.8 – 3728.8)	100.5 (51.0 – 172.1)	41.9 (22.4 – 74.8)
**Suriname**	8.8 (7.7 – 10.1)	104.8 (89.3 – 123.5)	2.1 (1.5 – 2.7)	1.4 (0.8 – 2.4)
**Sweden**	3024.8 (2545.5 – 356	39447.9 (33236.8 – 46301.1)	485.8 (424.4 – 533.3	620.9 (338.6 – 1091.
**Switzerland**	1933.8 (1673.0 – 222	25445.0 (21806.7 – 29143.4)	318.5 (264.6 – 358.9	387.1 (230.1 – 653.1
**Syrian Arab Republic**	336.6 (285.7 – 393.3	3450.0 (2926.7 – 4030.0)	187.2 (130.1 – 255.1	54.5 (33.2 – 90.0)
**Taiwan (Province of China)**	929.4 (746.6 – 1150.	9713.6 (7835.6 – 11848.0)	139.7 (129.4 – 148.8	142.9 (76.8 – 262.2)
**Tajikistan**	267.0 (217.2 – 324.8	3537.0 (2911.6 – 4276.6)	0.1 (0.1 – 0.2)	53.0 (28.0 – 92.2)
**Thailand**	1164.3 (955.1 – 138	12707.8 (10369.2 – 15358.9)	68.1 (42.9 – 142.0)	191.8 (99.8 – 326.2)
**Timor-Leste**	6.5 (5.4 – 8.0)	60.9 (49.6 – 74.6)	0.2 (0.1 – 0.9)	0.8 (0.4 – 1.5)
**Togo**	15.0 (12.9 – 17.1)	125.0 (102.3 – 149.0)	11.0 (6.0 – 16.3)	1.3 (0.7 – 2.4)
**Tokelau**	0.0 (0.0 – 0.1)	0.5 (0.4 – 0.6)	0.0 (0.0 – 0.0)	0.0 (0.0 – 0.0)
**Tonga**	1.7 (1.5 – 2.1)	19.1 (15.9 – 23.0)	0.3 (0.2 – 0.4)	0.3 (0.2 – 0.5)
**Trinidad and Tobago**	29.3 (25.2 – 33.6)	376.5 (317.5 – 441.9)	4.9 (4.4 – 5.5)	5.0 (2.5 – 8.8)
**Tunisia**	210.1 (182.6 – 240.3	2295.2 (1959.3 – 2671.3)	39.6 (25.9 – 56.3)	29.1 (15.6 – 50.7)
**Turkmenistan**	210.5 (171.6 – 254.5	2698.1 (2242.4 – 3270.4)	0.4 (0.4 – 0.5)	38.4 (20.2 – 66.0)
**Tuvalu**	0.2 (0.2 – 0.3)	2.4 (1.9 – 2.9)	0.1 (0.0 – 0.1)	0.0 (0.0 – 0.1)
**Türkiye**	2073.7 (1828.4 – 234	22594.4 (19386.5 – 26200.1)	626.0 (372.1 – 915.3	287.7 (149.4 – 523.2
**Uganda**	91.4 (80.5 – 103.2)	740.9 (614.1 – 876.4)	36.7 (16.7 – 63.6)	9.4 (4.9 – 17.0)
**Ukraine**	7753.8 (6848.3 – 869	113053.8 (101446.7 – 12482	30.0 (26.1 – 35.3)	1654.0 (908.7 – 2948
**United Arab Emirates**	20.5 (18.1 – 23.3)	202.3 (174.4 – 233.2)	7.2 (4.5 – 10.7)	2.1 (1.1 – 3.7)
**United Kingdom**	13897.9 (12151.6 – 1	180508.5 (158663.1 – 20287	2151.6 (1925.9 – 226	2888.7 (1618.0 – 507
**United Republic of Tanzania**	181.1 (160.9 – 203.1	1447.7 (1218.5 – 1700.2)	86.2 (48.2 – 134.7)	18.1 (9.2 – 32.9)
**United States of America**	95755.5 (85277.6 – 1	1279737.3 (1164442.8 – 140	9716.7 (8168.8 – 104	19617.4 (11114.9 – 3
**United States Virgin Islands**	5.2 (4.6 – 5.9)	61.5 (52.6 – 71.0)	1.2 (0.9 – 1.6)	0.8 (0.4 – 1.3)

**Uruguay**	448.1 (391.0 – 510.7	4897.9 (4250.8 – 5652.3)	113.6 (99.4 – 125.4)	84.0 (47.2 – 146.3)
**Uzbekistan**	1173.5 (964.5 – 1421	16355.0 (13509.9 – 19630.3)	12.2 (8.7 – 18.2)	251.6 (139.7 – 440.0
**Vanuatu**	1.6 (1.3 – 1.9)	15.2 (12.6 – 18.6)	0.3 (0.2 – 0.6)	0.2 (0.1 – 0.3)
**Venezuela (Bolivarian Republic of**	416.4 (353.0 – 487.9	5069.3 (4256.3 – 5997.2)	66.2 (59.6 – 73.2)	67.1 (34.8 – 119.1)
**Viet Nam**	1227.4 (1005.2 – 148	14511.8 (11787.0 – 17709.3)	45.9 (21.4 – 162.6)	236.1 (127.5 – 422.0
**Yemen**	188.2 (166.5 – 214.3	1861.9 (1584.8 – 2172.9)	52.2 (25.2 – 91.2)	23.8 (12.3 – 42.2)
**Zambia**	46.6 (41.0 – 52.8)	353.7 (296.7 – 418.4)	27.6 (15.3 – 41.5)	4.1 (2.1 – 7.4)
**Zimbabwe**	59.9 (52.2 – 68.3)	559.4 (465.7 – 662.7)	24.0 (13.4 – 37.4)	6.4 (3.3 – 11.6)

ountries and regions.

	2021			
**DALYs**	**Incidence**	**Prevalence**	**Deaths**	**YLDs**
2024.8 (800.5 – 3987	363.7 (318.7 – 415.6)	3282.2 (2776.	110.2 (48.8 – 203.0)	40.8 (21.2 – 70.3)
319.6 (221.4 – 471.1	588.8 (503.3 – 675.8)	8518.2 (7288.	39.7 (20.9 – 64.5)	113.0 (63.8 – 190.9)
3922.0 (2477.8 – 577	1731.0 (1498.6 – 1962.1)	19129.9 (162	287.2 (188.9 – 420.3)	239.4 (131.5 – 416.4)
5.0 (3.3 – 6.8)	2.0 (1.7 – 2.4)	22.3 (19.0 – 2	0.3 (0.2 – 0.4)	0.3 (0.2 – 0.6)
16.5 (10.7 – 24.1)	17.3 (14.9 – 20.1)	219.0 (186.2	3.1 (1.9 – 4.4)	2.8 (1.6 – 4.8)
1032.5 (537.6 – 1654	214.3 (191.0 – 243.5)	1687.8 (1415.	117.7 (72.2 – 189.1)	19.3 (10.0 – 35.4)
7.8 (7.0 – 8.7)	4.7 (4.0 – 5.4)	61.1 (50.5 – 7	0.6 (0.6 – 0.7)	0.8 (0.4 – 1.4)
14709.3 (12983.5 – 1	8649.8 (7385.2 – 10316.8)	100584.0 (85	1262.2 (1078.0 – 1418.5)	1765.5 (989.1 – 3057.8)
151.5 (121.5 – 191.9	632.8 (533.8 – 745.2)	8683.3 (7454.	17.0 (14.2 – 19.8)	128.8 (72.0 – 215.2)
7401.4 (6697.1 – 803	7463.9 (6423.8 – 8579.0)	88129.5 (752	954.5 (731.6 – 1088.2)	1240.0 (695.3 – 2066.5)
5656.7 (5079.3 – 626	6929.8 (5614.0 – 8567.4)	101546.4 (80	828.0 (650.4 – 935.7)	1469.8 (813.6 – 2483.3)
182.8 (126.0 – 272.3	1354.5 (1094.6 – 1599.2)	16013.4 (132	4.4 (2.7 – 6.3)	207.0 (111.8 – 363.8)
29.3 (25.7 – 32.6)	19.2 (16.4 – 22.4)	245.0 (206.1	2.7 (2.2 – 3.3)	3.1 (1.6 – 5.5)
60.5 (45.0 – 83.7)	45.6 (39.0 – 53.3)	431.6 (363.7	5.8 (4.0 – 8.0)	4.1 (2.1 – 7.2)
5900.5 (3027.1 – 101	3448.6 (2948.3 – 3977.7)	35206.0 (291	820.9 (508.1 – 1264.4)	503.8 (279.7 – 895.8)
35.2 (30.9 – 40.6)	23.2 (19.6 – 27.9)	335.1 (279.9	3.2 (2.6 – 3.8)	4.8 (2.6 – 8.2)
387.5 (240.8 – 641.5	2193.7 (1809.3 – 2642.8)	33441.5 (283	14.5 (12.0 – 17.4)	471.8 (261.3 – 816.8)
12179.2 (10519.2 – 1	4573.9 (4085.7 – 5066.5)	51512.8 (455	1262.7 (929.1 – 1457.7)	824.4 (471.4 – 1367.1)
9.8 (8.6 – 11.6)	10.4 (8.9 – 12.0)	128.4 (107.6	1.3 (1.2 – 1.5)	1.7 (0.9 – 3.0)
297.7 (160.6 – 450.1	66.7 (58.0 – 75.3)	586.0 (481.3	26.1 (14.7 – 43.8)	6.7 (3.7 – 12.3)
33.7 (29.4 – 38.0)	12.2 (10.1 – 14.8)	183.6 (150.2	2.6 (2.1 – 3.2)	2.4 (1.3 – 4.3)
30.6 (13.8 – 57.0)	17.4 (15.2 – 19.9)	185.4 (159.3	3.8 (2.4 – 5.5)	2.8 (1.4 – 5.1)
733.9 (371.8 – 1094.	396.3 (336.4 – 465.5)	4899.3 (4122	49.5 (26.0 – 72.9)	63.2 (34.2 – 109.7)
1047.4 (706.0 – 1565	1180.8 (1005.6 – 1364.8)	17321.6 (147	99.6 (58.2 – 142.9)	236.6 (131.4 – 406.0)
123.1 (78.3 – 189.2)	29.2 (25.6 – 33.0)	289.8 (243.4	7.4 (5.1 – 10.7)	3.3 (1.7 – 6.1)
33854.4 (32712.5 – 3	16538.6 (14179.6 – 19151.1)	206874.1 (17	2901.3 (2521.5 – 3133.3)	2924.3 (1575.7 – 5141.0)
37.5 (27.7 – 50.3)	81.5 (70.6 – 93.4)	820.5 (708.9	4.6 (2.9 – 6.0)	10.7 (5.8 – 18.6)
818.7 (680.7 – 1034.	1548.2 (1306.9 – 1824.1)	27477.5 (234	134.2 (110.5 – 165.5)	401.0 (223.6 – 681.4)
810.6 (363.2 – 1248.	119.0 (104.6 – 133.4)	1000.6 (823.5	73.2 (36.8 – 128.5)	11.7 (6.2 – 20.8)
813.4 (417.7 – 1398.	59.9 (52.7 – 67.5)	452.3 (373.8	27.5 (15.4 – 48.2)	5.4 (2.9 – 9.8)
42.3 (21.6 – 80.9)	6.9 (6.2 – 7.7)	72.7 (61.4 – 8	3.5 (2.0 – 6.6)	1.0 (0.5 – 1.7)
141.9 (60.5 – 494.3)	425.0 (348.1 – 518.4)	4496.8 (3666.	9.6 (4.0 – 36.1)	65.6 (33.6 – 116.0)
1201.0 (611.3 – 1822	180.9 (158.8 – 205.1)	1576.5 (1317.	102.3 (58.9 – 171.5)	17.4 (9.0 – 31.0)
10605.5 (9618.7 – 11	16384.6 (13947.1 – 19561.1)	206447.3 (17	1849.9 (1449.3 – 2086.8)	2733.9 (1510.6 – 4554.0)
325.6 (157.1 – 528.0	32.8 (29.1 – 37.4)	231.9 (192.6	19.5 (10.2 – 30.8)	2.6 (1.3 – 4.8)
522.3 (248.9 – 843.6	65.7 (57.4 – 75.3)	529.1 (438.5	36.5 (18.6 – 61.3)	5.5 (3.0 – 10.1)
1879.3 (1746.9 – 201	3370.2 (2894.2 – 3883.0)	42958.8 (368	222.8 (188.2 – 246.0)	643.9 (375.8 – 1096.6)
24999.2 (15772.5 – 3	101018.4 (94640.8 – 108047.5)	1182214.6 (1	1062.5 (736.1 – 1557.5)	16499.1 (9250.0 – 28388.
5776.7 (5500.5 – 608	4241.6 (3567.4 – 4892.0)	56281.6 (469	591.1 (496.0 – 694.2)	799.0 (448.4 – 1384.2)
58.9 (25.1 – 93.3)	7.2 (6.4 – 8.1)	61.4 (52.0 – 7	3.5 (2.2 – 5.7)	0.8 (0.4 – 1.5)
377.5 (191.5 – 585.2	49.0 (43.6 – 54.7)	384.9 (325.0	29.6 (18.1 – 41.9)	4.6 (2.4 – 8.3)
3.0 (1.9 – 4.8)	0.9 (0.7 – 1.1)	11.7 (9.8 – 13	0.1 (0.1 – 0.3)	0.2 (0.1 – 0.3)
289.4 (261.7 – 319.3	390.0 (332.7 – 464.5)	5273.1 (4449.	45.7 (39.0 – 51.5)	72.8 (42.0 – 126.7)
1615.5 (1425.8 – 185	2352.7 (2015.2 – 2793.4)	38471.8 (328	297.4 (253.9 – 339.6)	502.0 (284.7 – 887.1)
1646.3 (1443.2 – 176	860.3 (745.8 – 987.5)	12869.9 (111	148.9 (127.5 – 170.5)	174.1 (97.3 – 305.2)
829.3 (548.3 – 1223.	511.3 (455.3 – 565.2)	4741.1 (4111	86.9 (57.8 – 119.1)	66.5 (37.8 – 116.2)

1820.2 (1603.2 – 212	5181.7 (4438.5 – 5994.2)	84519.5 (731	549.2 (475.4 – 613.4)	1120.2 (635.5 – 1895.7)
767.4 (392.3 – 1210.	140.7 (121.0 – 159.9)	1237.2 (1012.	69.2 (36.7 – 118.3)	12.9 (7.0 – 23.4)
746.3 (420.5 – 1979.	1235.6 (1011.6 – 1507.9)	16118.6 (131	37.2 (17.6 – 130.0)	272.9 (147.4 – 468.2)
3623.1 (1889.6 – 541	572.0 (506.7 – 645.2)	4430.6 (3714.	346.3 (194.9 – 623.1)	53.6 (28.8 – 99.3)
3280.4 (2968.6 – 360	2374.4 (2057.8 – 2754.8)	29209.5 (248	390.3 (313.2 – 439.9)	423.7 (238.4 – 730.3)
34.7 (18.5 – 56.1)	8.4 (7.4 – 9.6)	70.2 (58.8 – 8	3.5 (2.1 – 5.7)	0.8 (0.4 – 1.4)
3.2 (2.3 – 4.0)	2.6 (2.1 – 3.0)	36.6 (30.2 – 4	0.2 (0.1 – 0.2)	0.5 (0.3 – 0.9)
532.8 (346.3 – 666.7	349.8 (296.8 – 411.6)	4614.1 (3874	37.0 (23.3 – 50.1)	65.3 (34.6 – 116.6)
1267.9 (1154.6 – 136	865.2 (778.4 – 950.4)	11986.6 (107	79.6 (63.8 – 97.8)	163.0 (88.4 – 283.5)
11680.0 (7190.9 – 16	3380.1 (2898.1 – 3922.5)	31996.1 (267	480.6 (327.0 – 662.7)	346.1 (184.1 – 623.2)
109.6 (79.5 – 141.0)	250.4 (209.8 – 296.9)	3637.7 (2976	8.7 (4.9 – 11.7)	55.4 (28.7 – 97.3)
55.3 (26.1 – 91.7)	11.7 (10.2 – 13.2)	96.6 (80.7 – 1	4.7 (2.5 – 7.8)	1.2 (0.6 – 2.1)
395.0 (181.5 – 659.2	44.3 (39.1 – 50.3)	342.4 (284.7	23.2 (14.1 – 36.6)	4.0 (2.1 – 7.4)
318.2 (274.0 – 379.2	772.7 (658.1 – 888.6)	12903.1 (109	78.5 (65.6 – 88.5)	202.6 (116.2 – 343.8)
68.4 (43.7 – 94.8)	10.7 (9.2 – 12.3)	102.9 (84.4 –	3.9 (2.3 – 5.8)	1.2 (0.6 – 2.2)
5000.7 (2129.0 – 831	517.7 (465.9 – 573.5)	4096.0 (3579	210.6 (132.0 – 330.7)	52.0 (28.0 – 95.7)
27.0 (16.2 – 37.3)	26.2 (21.7 – 31.7)	283.5 (232.2	0.9 (0.6 – 1.3)	4.0 (2.1 – 7.3)
4442.0 (3728.6 – 518	3260.2 (2798.1 – 3861.7)	44215.5 (373	633.0 (485.4 – 727.1)	639.1 (360.3 – 1101.9)
52164.9 (46503.1 – 5	24179.7 (21356.3 – 27382.6)	304229.1 (26	5803.2 (4504.3 – 6596.9)	4727.8 (2770.4 – 8146.1)
163.0 (95.1 – 237.3)	21.7 (19.3 – 24.3)	186.3 (156.9	10.3 (6.4 – 15.6)	2.4 (1.2 – 4.2)
72.1 (33.0 – 119.9)	12.4 (10.9 – 13.9)	107.3 (89.9 –	8.5 (5.0 – 13.9)	1.3 (0.7 – 2.3)
329.4 (238.0 – 478.9	2133.5 (1809.6 – 2437.4)	31895.3 (278	101.5 (85.5 – 115.9)	550.0 (320.7 – 894.6)
92270.9 (82251.0 – 1	42731.4 (37957.2 – 47770.4)	562542.2 (47	10314.8 (8065.6 – 11906	8371.6 (4860.8 – 14058.3
2097.0 (1174.2 – 294	239.6 (208.6 – 270.6)	2195.0 (1836	140.5 (88.0 – 226.3)	25.6 (13.7 – 43.9)
5409.6 (4857.5 – 579	4130.0 (3703.3 – 4610.2)	55723.1 (491	992.3 (804.2 – 1118.0)	876.7 (498.0 – 1530.9)
20.1 (9.2 – 30.5)	16.9 (14.6 – 19.6)	169.3 (145.4	1.1 (0.7 – 1.7)	2.4 (1.3 – 4.1)
8.0 (6.4 – 9.8)	3.8 (3.2 – 4.5)	50.9 (42.4 – 6	0.5 (0.5 – 0.6)	0.7 (0.4 – 1.3)
25.2 (18.3 – 32.5)	10.1 (8.4 – 11.8)	130.5 (109.1	1.0 (0.7 – 1.3)	2.2 (1.2 – 3.8)
270.4 (199.1 – 335.6	395.7 (337.6 – 460.2)	4916.1 (4064	29.8 (25.8 – 34.6)	67.8 (35.0 – 122.1)
700.6 (340.5 – 1111.	72.0 (64.0 – 80.6)	592.7 (496.3	41.1 (21.8 – 68.9)	6.7 (3.6 – 11.8)
130.6 (60.0 – 205.3)	10.0 (8.8 – 11.4)	79.1 (64.6 – 9	6.9 (4.1 – 10.7)	0.8 (0.4 – 1.4)
130.5 (106.5 – 155.6	28.4 (24.9 – 32.6)	337.2 (290.0	6.9 (5.3 – 8.9)	4.2 (2.3 – 7.4)
1142.2 (529.9 – 1995	226.5 (197.7 – 256.6)	2301.2 (1939	66.3 (35.7 – 106.2)	26.2 (13.0 – 47.2)
324.4 (192.5 – 441.2	268.6 (225.3 – 315.0)	3142.7 (2603	47.1 (26.3 – 65.9)	41.1 (20.1 – 71.0)
6447.3 (5865.4 – 706	5226.9 (4438.0 – 6128.8)	84963.3 (742	802.9 (702.9 – 905.2)	1175.3 (686.5 – 2020.4)
126.2 (110.9 – 138.9	98.4 (82.9 – 118.3)	1234.9 (1019	23.0 (17.7 – 26.5)	16.1 (9.4 – 27.9)
55424.8 (31858.7 – 8	31167.6 (28765.6 – 33913.2)	323128.5 (29	6888.8 (4670.1 – 8764.3)	4560.7 (2506.7 – 7932.9)
3295.0 (1543.4 – 109	7907.0 (7297.1 – 8643.6)	79491.9 (729	223.6 (91.8 – 886.6)	1121.2 (606.8 – 1935.9)
6392.9 (4581.4 – 831	3383.4 (3111.1 – 3682.9)	38365.1 (348	455.2 (322.4 – 627.3)	456.6 (254.7 – 785.3)
1787.1 (1200.2 – 248	738.1 (632.1 – 854.8)	8400.6 (7083	138.7 (89.0 – 202.7)	107.3 (60.9 – 178.2)
1385.2 (1275.1 – 149	1077.5 (939.0 – 1225.1)	13281.9 (114	137.6 (108.5 – 159.3)	169.6 (95.3 – 291.0)
2548.5 (2314.4 – 278	1749.0 (1410.9 – 1969.6)	18051.3 (142	419.4 (327.3 – 478.0)	273.7 (157.6 – 483.1)
30025.9 (27056.7 – 3	51726.5 (48482.0 – 55264.9)	846172.8 (79	4166.2 (3114.3 – 4750.2)	12147.7 (6850.3 – 20886.
102.3 (89.3 – 117.2)	95.6 (81.2 – 111.3)	1386.7 (1159	9.4 (7.3 – 11.7)	21.6 (12.1 – 37.7)
88066.6 (78690.2 – 9	79341.6 (70580.9 – 89212.6)	1318808.1 (1	15387.7 (10338.8 – 1839	21220.7 (11992.4 – 36403
321.0 (199.6 – 435.0	255.0 (217.7 – 294.4)	2895.8 (2428	22.3 (15.0 – 31.9)	29.7 (16.0 – 51.7)
607.7 (442.6 – 858.1	2704.9 (2247.7 – 3258.3)	34879.9 (293	53.7 (44.3 – 64.4)	461.7 (247.4 – 813.2)
1469.2 (877.5 – 2033	346.9 (313.8 – 383.9)	2993.6 (2622	145.8 (98.4 – 212.0)	39.1 (21.5 – 69.3)
32.4 (23.3 – 44.8)	2.7 (2.3 – 3.2)	27.3 (23.0 – 3	1.6 (1.0 – 2.3)	0.4 (0.2 – 0.6)
95.8 (87.6 – 103.2)	143.5 (123.3 – 167.6)	1545.7 (1312	8.1 (6.8 – 9.3)	13.1 (7.3 – 22.6)

113.5 (80.4 – 170.1)	584.5 (490.3 – 685.0)	7103.6 (5928.	6.0 (5.0 – 7.3)	96.3 (51.0 – 168.5)
76.0 (29.9 – 282.8)	141.2 (116.2 – 173.4)	1477.9 (1220.	4.2 (1.7 – 16.7)	22.0 (11.8 – 38.8)
329.7 (271.4 – 407.9	729.9 (613.1 – 847.4)	12871.8 (108	57.2 (49.1 – 65.5)	208.4 (118.6 – 348.7)
1418.9 (773.5 – 233	274.4 (241.0 – 313.0)	3642.9 (3132.	69.6 (45.5 – 90.4)	49.0 (27.4 – 86.2)
134.3 (81.0 – 195.8)	20.5 (17.9 – 23.5)	193.7 (159.9	8.9 (5.6 – 13.3)	2.4 (1.2 – 4.3)
234.0 (115.4 – 353.4	26.1 (22.7 – 29.8)	215.6 (177.9	15.2 (8.2 – 25.6)	2.3 (1.2 – 4.1)
524.3 (327.5 – 805.7	219.8 (192.5 – 252.0)	2441.5 (2116.	54.8 (29.5 – 94.7)	29.6 (16.3 – 50.6)
279.4 (221.4 – 368.4	941.0 (796.4 – 1140.8)	16508.6 (140	51.9 (43.9 – 59.4)	266.4 (150.9 – 445.9)
422.7 (389.8 – 456.1	259.6 (230.0 – 297.3)	3293.4 (2843.	56.2 (46.1 – 63.5)	47.0 (26.8 – 79.7)
2275.8 (1143.7 – 35	195.1 (174.0 – 219.9)	1499.6 (1273.	135.5 (85.0 – 200.4)	16.4 (8.6 – 29.7)
757.3 (369.8 – 1196.	112.3 (99.3 – 127.5)	907.9 (761.7	48.7 (28.1 – 81.2)	11.7 (6.3 – 20.5)
818.6 (482.4 – 1019.	1240.4 (1041.0 – 1497.4)	13393.2 (112	68.7 (53.1 – 102.9)	191.8 (100.1 – 331.8)
11.5 (5.4 – 23.4)	17.2 (14.7 – 20.2)	181.4 (154.1	1.3 (0.9 – 1.8)	2.7 (1.6 – 4.5)
972.2 (432.4 – 1594.	84.2 (73.0 – 97.0)	719.4 (584.7	56.4 (31.2 – 89.4)	7.9 (4.1 – 14.3)
109.7 (99.0 – 118.8)	92.6 (79.9 – 108.3)	1223.1 (1054.	19.4 (15.6 – 22.4)	17.0 (9.7 – 29.5)
5.7 (3.3 – 10.5)	1.2 (1.0 – 1.5)	11.4 (9.5 – 13	0.2 (0.1 – 0.4)	0.1 (0.1 – 0.2)
289.8 (160.5 – 428.2	27.4 (24.1 – 30.9)	253.3 (212.2	17.8 (11.1 – 28.6)	3.1 (1.6 – 5.5)
148.3 (136.6 – 163.3	79.1 (66.3 – 96.5)	979.9 (821.1	17.1 (15.1 – 18.6)	14.6 (7.9 – 25.0)
6037.0 (5832.2 – 62	6955.3 (5748.2 – 8210.8)	89358.8 (745	596.4 (507.1 – 680.3)	1208.3 (638.1 – 2139.2)
17.5 (10.3 – 30.6)	2.9 (2.5 – 3.5)	29.1 (24.2 – 3	0.5 (0.3 – 0.9)	0.4 (0.2 – 0.7)
13.6 (9.0 – 20.1)	9.1 (7.4 – 11.0)	133.2 (110.9	1.2 (0.7 – 1.8)	1.9 (1.0 – 3.3)
95.5 (61.1 – 158.9)	328.0 (273.3 – 394.5)	3747.0 (3139.	6.0 (3.8 – 9.2)	48.1 (24.9 – 82.6)
77.4 (55.9 – 108.0)	141.4 (116.9 – 164.0)	2100.5 (1751.	7.7 (4.6 – 10.8)	25.2 (14.1 – 42.8)
4527.3 (2689.0 – 71	1500.4 (1337.4 – 1677.9)	16714.9 (145	325.9 (216.0 – 482.3)	220.2 (120.3 – 381.9)
1289.7 (619.4 – 208	189.2 (167.9 – 213.7)	1472.9 (1220.	100.0 (52.0 – 186.1)	18.0 (9.1 – 33.5)
878.4 (401.3 – 2773.	1644.9 (1340.5 – 2025.6)	17968.8 (146	45.6 (19.5 – 188.6)	274.6 (144.1 – 469.0)
129.5 (83.6 – 173.4)	26.0 (22.5 – 29.6)	256.0 (213.0	9.2 (5.8 – 14.0)	3.1 (1.6 – 5.5)
2.1 (1.2 – 3.9)	0.2 (0.2 – 0.3)	2.6 (2.1 – 3.0	0.1 (0.0 – 0.1)	0.0 (0.0 – 0.1)
1154.8 (595.4 – 193	693.2 (599.3 – 791.6)	6831.6 (5737.	138.3 (84.0 – 206.4)	95.4 (50.5 – 171.2)
17582.3 (15556.1 –	4993.9 (4417.0 – 5607.6)	50075.7 (432	1983.2 (1543.5 – 2244.8)	662.2 (369.9 – 1151.6)
1947.0 (1741.2 – 21	2095.1 (1855.1 – 2347.7)	22619.6 (200	295.0 (235.4 – 334.4)	332.6 (183.7 – 571.5)
88.8 (69.6 – 123.8)	204.8 (173.8 – 238.6)	2668.3 (2237.	9.6 (5.5 – 12.6)	35.5 (19.6 – 61.0)
568.0 (248.7 – 994.0	90.7 (79.2 – 103.9)	755.5 (618.3	51.4 (23.1 – 96.3)	8.1 (4.2 – 14.6)
10129.1 (5307.4 – 1	1225.6 (1105.5 – 1349.9)	10943.1 (964	586.4 (333.2 – 920.5)	117.6 (62.8 – 209.2)
0.5 (0.4 – 0.9)	0.1 (0.1 – 0.1)	1.1 (0.9 – 1.3	0.0 (0.0 – 0.0)	0.0 (0.0 – 0.0)
210.4 (160.7 – 318.9	474.1 (401.0 – 556.6)	6509.3 (5464.	29.1 (14.1 – 41.5)	78.7 (44.1 – 140.5)
10.1 (6.1 – 14.8)	2.6 (2.1 – 3.0)	25.8 (22.0 – 3	0.5 (0.3 – 0.6)	0.3 (0.2 – 0.6)
5247.5 (4688.5 – 56	3336.7 (2998.9 – 3707.9)	40570.6 (366	552.3 (426.4 – 623.4)	588.8 (325.9 – 1019.2)
70.1 (38.7 – 110.3)	75.4 (65.4 – 86.1)	811.9 (689.3	3.6 (2.3 – 4.7)	8.0 (4.2 – 13.8)
6624.5 (3794.7 – 10	3431.4 (3146.6 – 3738.5)	31503.3 (283	754.8 (467.2 – 1170.6)	419.6 (237.8 – 746.3)
2.6 (1.5 – 4.2)	0.9 (0.8 – 1.1)	9.2 (7.8 – 11.	0.1 (0.1 – 0.2)	0.1 (0.1 – 0.2)
98.8 (65.9 – 143.2)	79.4 (67.9 – 92.6)	906.6 (756.2	6.9 (5.0 – 9.2)	9.8 (5.3 – 17.5)
139.0 (125.7 – 153.2	195.0 (163.7 – 228.4)	2760.0 (2309.	24.2 (19.1 – 29.1)	39.9 (22.0 – 70.3)
481.0 (203.8 – 1031.	120.6 (100.5 – 144.1)	1162.4 (958.8	26.1 (13.7 – 48.4)	15.0 (7.8 – 25.9)
623.4 (465.2 – 867.2	414.1 (354.6 – 479.1)	5086.2 (4247.	75.6 (49.1 – 102.8)	68.6 (36.2 – 118.7)
1093.1 (748.4 – 146	1216.2 (1015.7 – 1452.7)	17172.6 (141	83.4 (52.5 – 119.5)	229.4 (121.5 – 402.1)
624.3 (368.8 – 759.0	2423.3 (2230.9 – 2634.8)	26643.3 (245	69.3 (30.9 – 89.0)	396.7 (219.6 – 678.9)
3689.7 (3381.8 – 41	6940.5 (6349.8 – 7582.8)	98201.9 (902	1837.9 (1579.9 – 2022.6)	1408.8 (802.0 – 2395.6)
3488.2 (3246.7 – 37	2579.0 (2227.7 – 2831.3)	29845.7 (252	868.0 (681.4 – 985.1)	456.5 (255.8 – 792.4)
698.7 (647.9 – 756.0	410.3 (339.2 – 498.4)	6523.6 (5302.	62.3 (50.1 – 73.8)	101.8 (58.3 – 169.6)

36.3 (25.7 – 54.3)	48.3 (41.3 – 55.9)	440.8 (371.4	3.9 (2.5 – 5.9)	3.2 (1.7 – 5.9)
4504.3 (3166.5 – 674	14484.1 (12356.8 – 16711.2)	196171.2 (16	902.4 (485.6 – 1232.4)	2459.7 (1371.4 – 4222.8)
145.2 (102.7 – 208.6	755.3 (632.2 – 911.1)	11127.7 (952	10.2 (9.0 – 11.7)	168.1 (93.5 – 284.9)
4688.3 (4004.4 – 567	10039.6 (8240.7 – 11917.9)	178631.1 (14	328.7 (285.4 – 379.0)	2594.3 (1426.0 – 4346.0)
9149.7 (7134.6 – 124	41100.0 (36316.1 – 46812.4)	589366.1 (52	1339.3 (1190.4 – 1468.3)	8483.8 (4666.3 – 14709.6
1392.2 (690.2 – 2272	119.4 (106.4 – 135.0)	935.3 (787.0	48.3 (31.0 – 82.9)	11.9 (6.2 – 21.7)
10.3 (7.7 – 12.0)	3.7 (3.2 – 4.3)	44.2 (37.4 – 5	0.5 (0.4 – 0.6)	0.6 (0.3 – 1.0)
15.1 (13.1 – 16.9)	9.7 (8.1 – 11.0)	130.9 (108.5	1.3 (1.1 – 1.5)	1.9 (1.0 – 3.2)
10.7 (9.2 – 12.4)	5.2 (4.5 – 6.1)	69.2 (58.8 – 8	0.7 (0.6 – 0.8)	1.0 (0.5 – 1.7)
19.4 (12.7 – 32.4)	5.3 (4.5 – 6.2)	60.3 (51.5 – 7	1.0 (0.7 – 1.8)	0.9 (0.5 – 1.6)
10.0 (7.3 – 14.3)	9.4 (8.1 – 10.7)	127.4 (106.9	1.1 (0.6 – 1.8)	1.8 (1.0 – 3.2)
11.4 (5.8 – 18.4)	1.5 (1.3 – 1.7)	14.5 (11.9 – 1	0.8 (0.6 – 1.3)	0.2 (0.1 – 0.3)
1688.5 (1118.2 – 241	990.1 (867.0 – 1140.7)	9192.2 (7918	132.7 (82.7 – 189.4)	76.2 (38.3 – 136.5)
683.0 (344.6 – 1030.	86.9 (76.4 – 98.4)	798.2 (668.2	59.6 (33.0 – 103.4)	9.7 (5.3 – 16.9)
5154.9 (3967.8 – 778	4281.1 (3725.5 – 4913.3)	62388.1 (547	552.6 (324.9 – 715.6)	855.9 (467.8 – 1464.0)
7.8 (5.0 – 9.7)	6.5 (5.6 – 7.8)	74.5 (63.7 – 8	0.4 (0.3 – 0.6)	1.2 (0.6 – 2.0)
355.6 (170.6 – 552.9	43.2 (38.0 – 48.7)	363.4 (304.6	26.4 (14.2 – 43.5)	4.2 (2.3 – 7.5)
349.7 (315.0 – 394.0	1197.8 (1004.6 – 1414.0)	15863.7 (134	36.4 (29.2 – 41.1)	196.0 (109.8 – 330.2)
754.8 (573.1 – 1192.	1144.6 (981.7 – 1340.6)	17417.4 (151	95.4 (46.0 – 131.0)	229.9 (129.7 – 391.9)
1273.4 (1169.2 – 139	2029.3 (1801.9 – 2258.1)	31003.1 (276	433.4 (349.9 – 498.6)	417.2 (234.8 – 695.6)
16.2 (7.9 – 30.4)	10.8 (9.2 – 12.5)	109.2 (91.4 –	1.3 (0.8 – 2.4)	1.4 (0.7 – 2.5)
649.3 (241.7 – 1162.	77.4 (68.3 – 88.7)	547.0 (451.4	36.3 (14.9 – 67.6)	6.1 (3.0 – 11.3)
5597.4 (3578.9 – 663	828.5 (726.2 – 925.8)	8269.2 (7155	342.8 (229.1 – 449.4)	106.8 (57.7 – 195.6)
499.1 (230.6 – 862.7	47.2 (41.2 – 54.1)	380.7 (314.1	20.3 (10.4 – 34.3)	4.3 (2.3 – 8.0)
28939.0 (25904.2 – 3	15812.1 (14217.7 – 17629.7)	219762.7 (19	4143.8 (3092.4 – 4761.5)	3047.3 (1724.8 – 5196.9)
1086.0 (575.5 – 134	1873.7 (1563.5 – 2219.0)	21048.4 (176	51.1 (32.0 – 95.1)	306.3 (171.8 – 516.0)
3558.5 (1622.2 – 637	674.9 (588.7 – 769.8)	6808.1 (5764	174.2 (92.2 – 286.2)	87.3 (49.5 – 144.6)
61.4 (41.4 – 78.3)	28.5 (24.4 – 32.7)	368.0 (312.3	4.2 (2.6 – 5.6)	5.1 (2.8 – 8.8)
6918.5 (6195.5 – 763	5333.6 (4465.1 – 6311.8)	70975.8 (596	764.0 (576.7 – 887.3)	1011.9 (545.6 – 1752.9)
4647.3 (3973.3 – 523	3354.9 (2927.0 – 3902.5)	46702.6 (404	530.1 (391.3 – 616.5)	643.5 (361.2 – 1064.6)
6120.6 (4279.5 – 844	1000.9 (866.9 – 1164.2)	10441.9 (887	319.1 (219.2 – 482.3)	158.0 (93.2 – 257.6)
3254.0 (3053.5 – 346	3729.6 (3114.3 – 4383.3)	43089.5 (358	324.8 (263.8 – 369.8)	632.5 (338.1 – 1068.2)
57.5 (32.6 – 97.9)	563.7 (456.5 – 688.1)	6115.5 (5017	0.2 (0.1 – 0.4)	75.6 (40.3 – 138.1)
1915.3 (1290.3 – 361	4785.0 (4014.5 – 5697.2)	57988.3 (481	418.3 (226.4 – 591.1)	927.3 (509.5 – 1597.2)
8.2 (3.6 – 27.4)	26.3 (21.2 – 32.5)	290.3 (233.5	0.7 (0.3 – 2.8)	4.3 (2.2 – 7.7)
329.2 (167.3 – 482.4	49.3 (43.1 – 56.2)	433.8 (359.1	30.8 (16.7 – 53.4)	4.7 (2.5 – 8.5)
0.3 (0.2 – 0.6)	0.1 (0.0 – 0.1)	0.7 (0.6 – 0.9	0.0 (0.0 – 0.0)	0.0 (0.0 – 0.0)
7.4 (5.1 – 12.7)	2.8 (2.4 – 3.3)	34.9 (29.4 – 4	0.4 (0.2 – 0.8)	0.6 (0.3 – 1.0)
138.2 (125.1 – 152.7	74.5 (63.9 – 88.7)	1052.7 (902.1	9.7 (7.7 – 12.0)	14.9 (8.0 – 26.7)
1215.2 (824.9 – 1697	599.4 (528.6 – 686.3)	7286.9 (6251	93.7 (53.0 – 153.9)	94.6 (53.2 – 163.9)
51.5 (33.5 – 79.4)	516.5 (422.4 – 626.5)	6320.2 (5174	5.7 (4.3 – 7.5)	88.9 (49.4 – 153.8)
2.1 (1.2 – 3.6)	0.4 (0.3 – 0.5)	4.3 (3.7 – 5.0	0.1 (0.0 – 0.1)	0.1 (0.0 – 0.1)
17625.3 (10541.3 – 2	5479.1 (4868.9 – 6164.0)	65758.0 (570	1113.0 (784.6 – 1535.1)	849.3 (495.4 – 1441.2)
1032.0 (482.6 – 1767	243.2 (215.0 – 273.6)	2019.1 (1700	89.8 (52.1 – 158.1)	26.0 (14.3 – 47.8)
2274.7 (1497.0 – 357	9216.9 (8251.7 – 10484.1)	140820.6 (12	164.2 (114.5 – 224.8)	2147.2 (1196.4 – 3824.5)
238.3 (147.2 – 359.7	164.0 (139.4 – 196.8)	1419.1 (1198	29.9 (21.6 – 42.4)	8.2 (3.5 – 15.9)
34496.9 (31946.2 – 3	26109.8 (23365.3 – 29044.8)	328706.5 (29	3828.9 (3084.1 – 4179.3)	4736.2 (2612.1 – 8182.5)
2438.0 (1360.4 – 366	451.7 (401.3 – 505.9)	3686.4 (3106	195.4 (123.5 – 330.6)	47.0 (24.7 – 89.3)
162564.6 (145102.8	195159.3 (178657.1 – 212465.1	2415480.6 (2	16496.5 (12810.6 – 1837	34805.1 (20285.9 – 58582
34.7 (24.7 – 46.6)	13.7 (11.7 – 15.8)	200.8 (174.6	1.2 (0.8 – 1.8)	3.0 (1.7 – 5.0)

1866.3 (1667.1 – 203	1159.3 (1002.2 – 1346.5)	13878.2 (117	276.7 (229.2 – 308.2)	262.0 (150.2 – 440.9)
636.3 (479.3 – 854.5	3886.6 (3250.7 – 4691.4)	44403.0 (369	66.7 (53.1 – 81.7)	603.6 (330.0 – 1029.7)
13.7 (7.2 – 26.1)	5.3 (4.5 – 6.3)	53.6 (44.6 – 6	0.9 (0.6 – 1.8)	0.7 (0.4 – 1.2)
1870.3 (1711.2 – 203	1686.4 (1418.1 – 1986.0)	22106.8 (184	226.7 (176.2 – 292.9)	314.2 (178.1 – 558.8)
1155.2 (627.6 – 3336	3512.1 (2936.9 – 4227.5)	40384.6 (334	99.9 (41.3 – 394.4)	614.4 (321.9 – 1036.5)
1643.8 (778.5 – 3018	530.7 (471.5 – 595.6)	5180.4 (4448.	144.1 (79.2 – 228.6)	67.1 (35.7 – 117.0)
961.3 (471.9 – 1487.	134.8 (120.1 – 150.3)	1039.2 (878.5	66.5 (42.6 – 104.4)	12.9 (6.8 – 24.0)
693.1 (356.2 – 1247.	115.0 (101.1 – 131.6)	1066.6 (885.9	57.2 (36.5 – 81.8)	11.5 (5.8 – 21.2)

**DALYs**
4208.5 (1701.1 – 7857.0)
747.1 (441.5 – 1147.7)
8088.1 (5400.7 – 12121.8)
8.5 (5.4 – 11.2)
41.0 (26.9 – 57.6)
3258.7 (2044.6 – 5114.6)
15.3 (13.9 – 16.6)
19552.2 (17301.4 – 21665.3)
420.8 (344.6 – 523.6)
12106.2 (9752.1 – 13615.8)
10690.1 (8902.5 – 12044.7)
311.6 (209.5 – 460.5)
74.9 (60.6 – 93.7)
168.7 (115.8 – 236.3)
17707.2 (11094.1 – 25963.1)
66.1 (54.4 – 80.0)
732.0 (521.7 – 1095.4)
14670.6 (11452.0 – 16719.2)
38.8 (34.5 – 44.2)
661.7 (374.8 – 1083.7)
42.9 (35.7 – 53.2)
77.9 (50.1 – 116.0)
1375.8 (779.5 – 2061.0)
1861.5 (1176.3 – 2590.9)
194.5 (130.6 – 303.0)
63551.0 (57870.2 – 67558.8)
103.2 (67.2 – 130.4)
2650.3 (2165.8 – 3217.3)
1791.3 (912.3 – 3030.9)
885.9 (522.0 – 1454.5)
65.5 (38.0 – 120.5)
294.8 (147.5 – 961.1)
2863.7 (1636.4 – 4592.8)
23606.9 (19752.9 – 26660.7)
563.7 (291.6 – 900.3)
1059.3 (546.9 – 1751.4)
4124.9 (3633.0 – 4653.2)
37094.5 (26912.2 – 52184.7)
13809.3 (11797.2 – 16054.3)
92.6 (58.4 – 142.8)
809.5 (489.5 – 1174.7)
2.6 (1.7 – 5.2)
1036.1 (907.4 – 1173.6)
4631.5 (3970.9 – 5317.9)
3281.9 (2815.5 – 3774.2)
1203.8 (851.7 – 1601.8)

8933.8 (7847.4 – 10110.6)
1946.1 (1037.1 – 3285.5)
1086.2 (604.1 – 3157.8)
8900.4 (5061.9 – 15077.3)
5042.1 (4308.1 – 5564.0)
98.6 (58.6 – 166.2)
4.1 (2.8 – 5.3)
1036.9 (678.9 – 1365.9)
2132.6 (1684.3 – 2651.8)
16541.8 (10505.0 – 21032.0)
254.7 (164.2 – 333.6)
123.0 (63.8 – 207.7)
690.3 (415.6 – 1077.2)
1278.4 (1090.3 – 1477.1)
120.4 (69.1 – 191.3)
6370.4 (4057.2 – 9930.1)
33.7 (21.5 – 46.8)
7847.8 (6421.1 – 8892.1)
64054.2 (51558.2 – 72618.6)
235.6 (138.8 – 347.7)
218.4 (127.3 – 345.8)
2346.6 (2009.9 – 2800.5)
124470.5 (99862.9 – 141137.5)
3520.3 (2216.3 – 5435.1)
12297.5 (10226.3 – 13770.8)
22.1 (15.4 – 32.2)
13.2 (11.5 – 15.0)
28.7 (20.8 – 37.2)
863.2 (742.8 – 1005.9)
1120.9 (605.2 – 1796.9)
217.6 (128.2 – 341.5)
205.1 (155.7 – 271.0)
2298.0 (1165.3 – 3796.9)
1194.9 (711.0 – 1678.4)
13348.0 (11827.0 – 14914.1)
289.1 (239.3 – 329.5)
158556.3 (107622.7 – 204820.5)
6477.1 (3155.0 – 22162.5)
11727.7 (8911.7 – 16841.8)
4373.9 (2880.3 – 6150.8)
1900.6 (1586.1 – 2153.5)
5265.0 (4385.4 – 5886.3)
60325.8 (48529.0 – 69682.4)
233.1 (181.1 – 295.6)
169461.7 (123532.0 – 199413.8)
659.5 (455.5 – 918.0)
1707.9 (1390.7 – 2114.7)
3820.0 (2657.2 – 5517.1)
59.1 (39.0 – 85.2)
258.9 (223.6 – 297.2)

258.0 (204.1 – 328.0)
135.3 (63.9 – 493.9)
1076.8 (924.0 – 1271.0)
1424.0 (1016.1 – 1865.6)
256.0 (155.8 – 391.9)
455.2 (235.7 – 725.0)
1849.4 (1052.1 – 2957.8)
1059.5 (894.3 – 1277.0)
713.2 (606.4 – 793.7)
4938.7 (2985.4 – 7502.3)
1421.7 (816.1 – 2225.9)
1646.8 (1279.4 – 2380.4)
29.1 (20.1 – 37.4)
1800.1 (954.5 – 2820.3)
268.9 (226.1 – 306.1)
10.3 (5.3 – 18.7)
428.9 (274.0 – 663.5)
350.2 (316.4 – 375.0)
14957.9 (12860.1 – 17328.3)
17.7 (10.9 – 30.6)
16.6 (10.3 – 24.5)
209.0 (140.4 – 296.4)
153.2 (102.9 – 205.8)
9227.1 (6059.4 – 13890.3)
2788.5 (1465.2 – 4948.3)
1317.6 (661.4 – 4560.4)
233.4 (139.8 – 400.6)
2.5 (1.4 – 4.7)
3191.8 (1970.1 – 4649.8)
22988.1 (18666.0 – 25686.4)
3858.1 (3212.7 – 4324.2)
284.3 (176.9 – 363.6)
1445.2 (657.2 – 2604.9)
14804.0 (7845.7 – 23800.8)
0.4 (0.3 – 0.8)
580.0 (324.7 – 803.7)
13.1 (9.0 – 16.5)
6337.9 (5256.5 – 7146.5)
114.5 (79.3 – 150.8)
20846.8 (12097.7 – 34280.1)
3.3 (2.2 – 6.3)
196.6 (145.4 – 262.4)
574.3 (470.8 – 684.4)
1243.3 (621.4 – 2268.2)
1753.5 (1164.8 – 2386.5)
2301.8 (1504.5 – 3243.5)
2063.6 (1144.2 – 2656.1)
28482.3 (25077.6 – 31281.9)
10711.6 (8750.9 – 11988.6)
1155.5 (972.2 – 1360.6)

122.9 (75.0 – 185.8)
13271.5 (8365.2 – 17484.8)
372.5 (292.8 – 495.2)
8050.7 (6559.6 – 9910.9)
33868.6 (29464.5 – 40158.0)
1311.7 (845.5 – 2131.5)
12.1 (10.0 – 14.4)
27.7 (23.3 – 33.4)
18.6 (16.1 – 21.4)
29.1 (19.0 – 52.4)
15.1 (9.2 – 22.3)
21.0 (13.8 – 33.6)
5102.0 (3123.4 – 7277.6)
1463.4 (841.5 – 2407.8)
9547.2 (6089.2 – 12146.7)
9.1 (6.8 – 13.1)
732.6 (390.9 – 1165.7)
686.6 (576.3 – 836.8)
1805.8 (996.6 – 2449.9)
5481.9 (4532.7 – 6236.8)
46.7 (27.1 – 86.0)
1220.7 (502.1 – 2163.9)
7776.2 (5411.6 – 10572.7)
651.0 (329.5 – 1074.4)
49678.4 (39278.1 – 56426.9)
1305.0 (877.1 – 2275.6)
6231.0 (3109.5 – 10698.2)
113.9 (73.9 – 150.6)
9077.3 (7207.5 – 10564.2)
6418.2 (5082.1 – 7349.2)
8799.2 (5989.9 – 13650.7)
5406.9 (4698.9 – 6025.5)
82.8 (47.7 – 145.7)
8390.5 (4730.8 – 11591.6)
22.4 (10.6 – 75.6)
829.6 (459.7 – 1391.0)
0.4 (0.2 – 0.6)
9.3 (6.1 – 18.8)
245.2 (194.1 – 305.1)
2365.5 (1438.5 – 3943.7)
254.5 (196.1 – 334.8)
2.2 (1.4 – 3.9)
24819.7 (17468.3 – 33891.3)
2527.4 (1475.8 – 4263.6)
5040.9 (3677.4 – 6940.5)
931.8 (688.2 – 1368.7)
50040.0 (42993.3 – 55056.2)
5062.6 (3152.0 – 8661.2)
232320.7 (196312.1 – 260866.5)
26.1 (17.7 – 39.6)

3728.5 (3215.7 – 4150.3)
2430.7 (1948.8 – 3002.4)
35.2 (19.4 – 67.0)
5645.1 (4352.5 – 7350.7)
2372.1 (1204.6 – 7713.1)
4623.8 (2464.9 – 7718.9)
2013.8 (1267.6 – 3131.2)
2003.5 (1233.0 – 3114.2)

Supplementary Materials S5. Joinpoint Model

Non-rheumatic valvular heart disease							
	APC					AAPC	P value
	Segment1	Segment2	Segment3	Segment4	Segment5		
Global	-0.2233*	0.2956*	0.8740*	-0.1686*	-1.0441*	0.1101*	<0.0001
Low SDI	-0.3286*	0.0576*	0.3522*	0.7418*	0.4117*	0.1339*	<0.0001
Low-middle SDI	-0.0617*	0.3071*	0.5440*	1.1471*	0.5068*	0.3144*	<0.0001
Middle SDI	0.2357*	0.6899*	2.0205*	0.7527*	-0.8374*	0.5773*	<0.0001
High-middle SDI	0.4242*	0.6399*	1.2404*	0.2266*	-0.9402*	0.3971*	<0.0001
Hgh SDI	0.1187	1.0637*	1.2456*	0.1104*	-0.7637*	0.5675*	<0.0001
**Non-rheumatic calcific aortic valve disease**							
	APC					AAPC	P value
	Segment1	Segment2	Segment3	Segment4	Segment5		
Global	0.3938*	1.5519*	0.2872*	-0.1676*	-0.8375*	0.4883*	<0.0001
Low SDI	0.0076	0.2975*	0.7112*	1.2850*	0.2263	0.5745*	<0.0001
Low-middle SDI	0.9732*	0.5620*	1.2320*	2.0480*	-0.2441	0.9993*	<0.0001
Middle SDI	1.3380*	2.0445*	1.2317*	1.9825*	0.0797	1.4615*	<0.0001
High-middle SDI	1.3626*	2.8439*	1.7236*	0.8002*	-0.9434*	1.0685*	<0.0001
Hgh SDI	0.5450*	0.9590*	1.9376*	0.5862*	0.1683*	0.8769*	<0.0001
**Non-rheumatic degenerative mitral valve disease**							
	APC					AAPC	P value
	Segment1	Segment2	Segment3	Segment4	Segment5		
Global	-0.6354*	-0.0002	0.4707*	-0.3924*	-1.9381*	-0.2823*	<0.0001
Low SDI	-0.7425*	-0.5065*	-0.2279*	-0.1078*	0.2936*	-0.1886*	<0.0001
Low-middle SDI	-0.4921*	-0.2143*	0.0743*	0.3602*	0.6565*	-0.1372*	<0.0001
Middle SDI	-0.3373*	-0.0601	2.1053*	0.3214*	-1.3572*	0.0695*	<0.0001
High-middle SDI	-0.4379*	0.0416	1.1241*	-0.123	-0.8856*	-0.1530*	<0.0001
Hgh SDI	-0.3015*	1.0202*	0.3880*	-0.0151	-1.7205*	0.1975*	<0.0001

* Indicates that APC or AAPC is significantly different from zero at the alpha = 0.05 level

Supplementary Materials S6. Data used for APC model

	1990-1991	1990-1991p	1992-1996	1992-1996p	1997-2001
**<5**	0	300633548.8	0	298257678.5	0
**5-9**	0	285958684.5	0	295888583	0
**10-14**	0	262883295.7	0	276697832.7	0
**15-19**	410.5931	255410765.4	427.4880255	256868886.8	432.4198676
**20-24**	1197.001	245158519.1	1249.162118	249785247.2	1217.227487
**25-29**	1931.795	223304675.4	2016.590765	237643771.3	1965.933516
**30-34**	2527.934	191451036.5	2726.358972	210608626.6	2646.863767
**35-39**	2965.988	175556364.2	3255.628856	185118771.4	3250.661787
**40-44**	5038.762	143717645.5	5601.082133	162773757.6	5775.517887
**45-49**	7464.966	114574067.6	8594.500711	129949476.7	9567.061664
**50-54**	18231.26	105490118.8	18329.21149	107765129.3	20163.01382
**55-59**	33190.09	93000345.25	34861.55455	98417976.04	34864.45939
**60-64**	65438.35	82758145.78	66114.11341	86466329.04	72371.42194
**65-69**	102032.4	67194973.67	108367.9244	72947039.71	119727.3468
**70-74**	78306.32	47875706.2	92499.53198	54871482.73	110858.0785
**75-79**	43997.71	36329074.44	44115.03553	36753443.12	57051.11062
**80-84**	20650.61	22441012.86	23073.08202	24387619.96	23696.52556
**85-90**	8483.761	10269177.07	9642.007863	11798593.87	10222.89771
**90-94**	2928.533	3131650.186	3326.347095	3845918.182	3258.936156
**>95**	999.3607	773225.8404	1076.489768	909573.2278	996.3049909

*The even columns represent age-stratified data for female NRVHD, while the odd columns represent gl

1997-2001p	2002-2006	2002-2006p	2007-2011	2007-2011p	2012-2016
294193688.5	0	298653225.8	0	313154649.1	0
292371755	0	288207706.6	0	294380433.8	0
298087905.6	0	296550495.6	0	291686954.7	0
273803767.3	457.0607293	299443892.2	468.554	299750225.5	469.4545
253638151.1	1306.868613	272158756	1424.624	300035347.2	1453.868
247298142.2	2040.045424	252305176.6	2258.362	271527133.4	2449.566
235166247	2764.81763	245715440.9	2960.306	251369887.7	3268.837
207529074.8	3413.397365	232611114.5	3676.931	243584780.8	3927.544
179186811.3	6267.303128	201725380.1	6767.599	227341145.7	7238.044
156496940.6	10476.43038	173295710.3	11560.03	196602234.4	12587.3
123900553.2	23879.7857	150918471.3	26555.73	168738142.6	29253.98
102469603.8	40430.9002	119311596.2	48557.74	146906842	54228.21
93701404.41	79452.75353	98496133.35	97107.61	116136575	116419.7
78716050.81	137518.2921	86217744.01	155492.6	91670154.77	186163.1
63061120.76	123536.5931	68877870.77	144173.3	76613675.38	158256.7
43748249.56	67511.92075	51035951.87	77763.72	56835805.7	90769.17
25598787.47	30897.87165	31350418.86	38859.16	37388672.91	45636.72
13725622.25	11513.43447	14905516.19	16183.21	19020251.05	20113
4901146.419	4081.379127	5907069.552	5481.509	6790583.43	7544.441
1226917.633	1332.862464	1667563.686	2024.362	2162261.989	2776.948

lobal female population age-stratified data.

2012-2016p	2017-2021	2017-2021p
324618172.1	0	325892752.7
312112782.6	0	327204972
297165916.7	0	315270318.3
293855173.2	482.3377	298458927.9
299267079.6	1450.809	293363559.7
297948024.5	2486.467	295411275.6
269791770.1	3523.73	295074995.9
248767725	4312.84	266657616.8
238377670.4	7712.772	243570870.7
222395698.9	13577.01	233516065.6
192241471.4	31293.94	217654314.2
164763279.9	58673.14	187658903.5
143444529.3	126095.5	160290734.8
109217887.1	215824.3	135242676.7
82468263.41	187032.4	98998479.81
64395624.49	102678	70044895.7
42464972.99	55390.7	48828643.63
23250413.19	23544.34	26965028.82
9074090.206	9065.601	11308500.57
2671984.962	3562.34	3567131.571

Supplementary Materials S7. Data used for Decomposition Analysis

	location	Overll difference	Aging	Population	Epidemiol	a_percent	p_percent	r_percent
1	Global	487191.4	169843	296998.2	20350.22	34.86	60.96	4.18
2	High SDI	248641.94	92798.99	90251.21	65591.74	37.32	36.3	26.38
3	High-midd	121896.2	54230.83	47003.93	20661.43	44.49	38.56	16.95
4	Middle SD	81743.6	32808.57	35791.3	13143.74	40.14	43.78	16.08
5	Low-midd	28561.1	6691.542	18986.63	2882.935	23.43	66.48	10.09
6	Low SDI	5883.54	-183.156	5858.768	207.933	-3.11	99.58	3.53

	location	Overll difference	Aging	Population	Epidemiol	a_percent	p_percent	r_percent
1	Global	288801.42	90300.21	150970.2	47531.04	31.27	52.27	16.46
2	High SDI	164252.26	56094.33	52380.03	55777.9	34.15	31.89	33.96
3	High-midd	70398.38	24773.94	21156.59	24467.85	35.19	30.05	34.76
4	Middle SD	37304.41	12098.08	13159.91	12046.43	32.43	35.28	32.29
5	Low-midd	13807.4	2758.721	7439.473	3609.202	19.98	53.88	26.14
6	Low SDI	2684.15	-49.338	2242.986	490.507	-1.84	83.56	18.27

	location	Overll difference	Aging	Population	Epidemiol	a_percent	p_percent	r_percent
1	Global	198389.98	79542.75	146028.1	-27180.8	40.09	73.61	-13.7
2	High SDI	84389.68	36704.66	37871.18	9813.843	43.49	44.88	11.63
3	High-midd	51497.81	29456.89	25847.34	-3806.42	57.2	50.19	-7.39
4	Middle SD	44439.19	20710.49	22631.39	1097.306	46.6	50.93	2.47
5	Low-midd	14753.71	3932.821	11547.15	-726.267	26.66	78.27	-4.92
6	Low SDI	3199.39	-133.818	3615.782	-282.574	-4.18	113.01	-8.83

val_1990	val_2021	diff
391872.7	879064.1	487191.4
229238.2	477880.1	248641.9
104476	226372.2	121896.2
37331.63	119075.2	81743.6
16273.18	44834.28	28561.1
4084.566	9968.111	5883.545

val_1990	val_2021	diff
185018.3	473819.7	288801.4
125078.7	289330.9	164252.3
41166.19	111564.6	70398.38
11519.19	48823.6	37304.41
5584.695	19392.09	13807.4
1436.63	4120.785	2684.155

val_1990	val_2021	diff
206854.4	405244.4	198390
104159.5	188549.2	84389.68
63309.84	114807.7	51497.81
25812.45	70251.63	44439.19
10688.48	25442.19	14753.71
2647.937	5847.327	3199.39

Supplementary Materials S8. Predicted Incidence Data till 2046

	NRVHD		NRAVD		NRMVD	
**year**	**number**	**ASR**	**number**	**ASR**	**number**	**ASR**
**1990**	391872.7	21.63	185018.3	10.38	206854.4	11.26
**1991**	399718.1	21.56	189855.9	10.4	209862.2	11.16
**1992**	408057.5	21.52	194898.2	10.44	213159.2	11.08
**1993**	416695	21.49	200057.5	10.48	216637.6	11.01
**1994**	425155.3	21.45	205221.5	10.51	219933.9	10.94
**1995**	433410.9	21.41	210481.7	10.54	222929.3	10.87
**1996**	443061.8	21.42	216145	10.58	226916.8	10.84
**1997**	454633.6	21.5	222297.1	10.63	232336.5	10.87
**1998**	466926.3	21.61	228652.6	10.67	238273.7	10.93
**1999**	478672.5	21.68	234991.7	10.71	243680.9	10.97
**2000**	489269.6	21.7	241390	10.75	247879.6	10.95
**2001**	500826.8	21.73	249063.8	10.83	251763.1	10.91
**2002**	515218.9	21.88	258668.1	10.99	256550.8	10.89
**2003**	531088.1	22.09	269374	11.18	261714.1	10.9
**2004**	547229.1	22.28	280317.6	11.37	266911.4	10.9
**2005**	562311.9	22.41	290510.2	11.52	271801.7	10.89
**2006**	578560.7	22.54	301273.6	11.66	277287	10.88
**2007**	599413.1	22.79	314168.5	11.86	285244.6	10.94
**2008**	621682.5	23.08	327824.2	12.06	293858.3	11.02
**2009**	643696.7	23.3	341020.8	12.23	302675.9	11.07
**2010**	662336	23.39	352195.7	12.3	310140.3	11.09
**2011**	679448.5	23.35	362260.2	12.31	317188.3	11.04
**2012**	700216.6	23.38	373720.7	12.34	326495.9	11.03
**2013**	721327	23.4	385388.3	12.38	335938.7	11.02
**2014**	742999.3	23.37	396887.3	12.37	346112.1	10.99
**2015**	763685.2	23.32	407864.8	12.36	355820.4	10.96
**2016**	784554.8	23.21	418896.8	12.32	365658	10.89
**2017**	806581.3	23.12	431254.7	12.3	375326.5	10.82
**2018**	829098.4	23.04	443936	12.29	385162.3	10.75
**2019**	851212.1	22.92	455810.2	12.24	395401.9	10.68
**2020**	867574.9	22.68	464488.9	12.13	403086.1	10.55
**2021**	879064.1	22.36	473819.7	12.05	405244.4	10.31
**2022**	915012.7	22.48	492538.4	12.08	421462.3	10.38
**2023**	936794.4	22.37	505355.3	12.04	430045.2	10.3
**2024**	958746.8	22.26	518681.8	12	438258.4	10.22
**2025**	980258.2	22.14	531878.8	11.95	446360.1	10.15
**2026**	1001669	22.02	545173.8	11.9	454264.8	10.07
**2027**	1022868	21.9	558387.9	11.85	462042.5	10
**2028**	1044195	21.78	571830.5	11.8	469714.7	9.92
**2029**	1065814	21.66	585701.7	11.75	477234.2	9.85
**2030**	1087852	21.55	599685.9	11.7	485670.4	9.8
**2031**	1109518	21.43	613526.4	11.64	493905.1	9.75
**2032**	1130498	21.32	626966.6	11.59	501880	9.69
**2033**	1151110	21.21	640224.9	11.54	509694.4	9.64
**2034**	1171636	21.09	653547.6	11.48	517375.7	9.59
**2035**	1193315	21.01	667082.3	11.43	526744.3	9.57

**2036**	1214229	20.92	680304.8	11.39	535708.3	9.56
**2037**	1234047	20.84	693039.7	11.34	544109.5	9.54
**2038**	1252789	20.76	705455.3	11.29	551804	9.53
**2039**	1270748	20.67	717754.8	11.25	558876	9.51
**2040**	1289930	20.62	730438.2	11.21	567000.3	9.52
**2041**	1308536	20.56	742803.1	11.17	574915.7	9.52
**2042**	1326659	20.51	754756.1	11.13	582809.5	9.53
**2043**	1344370	20.45	766481	11.09	590574.1	9.54
**2044**	1361700	20.4	778115	11.06	598109.1	9.54
**2045**	1378712	20.34	789522.3	11.02	605606.6	9.55
**2046**	1395483	20.29	800587	10.98	613253.8	9.56

Supplementary Materials S9. Formulas for APC and AAPC

APC (Annual Percent Change) is a measure used to describe the percentage change in a time series dataset over a specific period, assuming a const

APC is calculated based on a log-linear model, assuming that the data changes linearly on a logarithmic scale. The formula for APC is:

$$APC=100\times(e^{\wedge \beta_1} \quad 1)$$

where β_1 is the slope in the regression model.

APC is mainly used to assess the internal trend within each segment of a segmented function or to evaluate the overall trend when no joinpoints are p

AAPC (Average Annual Percent Change) is a summary measure that provides an average percentage change over the entire study period. It is calcu

$$AAPC=100\times[e^{\wedge}(\sum w_i\beta_i / \sum w_i) \quad 1]$$

where w_i is the width of each segment interval (i.e., the number of years in the interval), and β_i is the regression coefficient for each interval.

The advantage of AAPC is that it can integrate trends from multiple time periods, especially when trends change significantly, providing an overall aver

ant rate of change. For example, if the APC is 1% and the incidence rate in a given year is 50/100,000, the incidence rate in the following year would be [illegible]

resent.

lated by taking a weighted average of the APCs from different segments of a segmented regression model, providing a more comprehensive reflection

rage rate of change.

$50 \times 1.01 = 50.5/100,000$.

of the overall trend. **The formula for AAPC is:**

Supplementary Materials S10. Temporal Changes in Mortality to Incidence Ratios Across Different SDI Regio

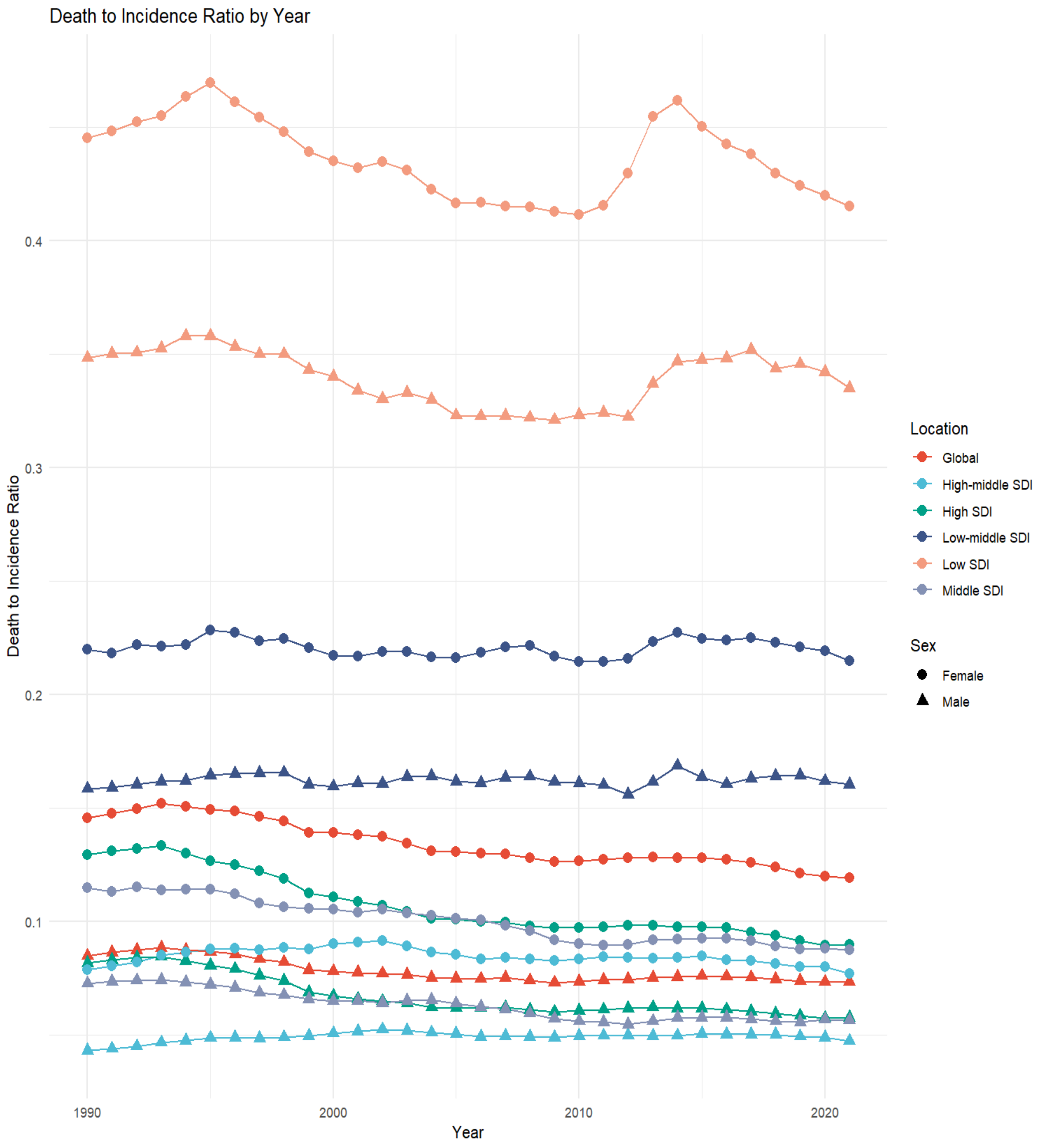

	location	year	sex	deaths	Mortality	Mortality_to_incidence_ratio
1	Global	1990	Female	2.65661	18.25849	0.145500053
2	Global	1990	Male	2.54879	30.04474	0.084833074
3	Global	1991	Female	2.68793	18.20073	0.1476824
4	Global	1991	Male	2.58189	29.92207	0.086286984
5	Global	1992	Female	2.71757	18.16214	0.149628378
6	Global	1992	Male	2.60831	29.82778	0.087445754
7	Global	1993	Female	2.7584	18.13399	0.152112124
8	Global	1993	Male	2.63145	29.74997	0.088452186
9	Global	1994	Female	2.72471	18.10063	0.150531197
10	Global	1994	Male	2.59224	29.66158	0.08739379
11	Global	1995	Female	2.69597	18.06789	0.14921333

12	Global	1995	Male	2.56202	29.58326	0.08660382
13	Global	1996	Female	2.68554	18.0745	0.14858171
14	Global	1996	Male	2.53633	29.61714	0.085637198
15	Global	1997	Female	2.65103	18.14031	0.146140472
16	Global	1997	Male	2.49006	29.81647	0.083512748
17	Global	1998	Female	2.62762	18.22453	0.144180594
18	Global	1998	Male	2.46824	30.07168	0.082078591
19	Global	1999	Female	2.54043	18.28152	0.138961594
20	Global	1999	Male	2.37764	30.25795	0.07857897
21	Global	2000	Female	2.54301	18.29935	0.138967307
22	Global	2000	Male	2.36581	30.32623	0.078012116
23	Global	2001	Female	2.52954	18.33087	0.137993424
24	Global	2001	Male	2.34969	30.32656	0.077479586
25	Global	2002	Female	2.53327	18.45418	0.137273424
26	Global	2002	Male	2.34338	30.42361	0.077025152
27	Global	2003	Female	2.50264	18.62571	0.134364709
28	Global	2003	Male	2.34158	30.58378	0.076562802
29	Global	2004	Female	2.46286	18.7851	0.131106935
30	Global	2004	Male	2.3148	30.7503	0.075277394
31	Global	2005	Female	2.46914	18.89486	0.130677923
32	Global	2005	Male	2.31707	30.91921	0.074939598
33	Global	2006	Female	2.46699	18.99696	0.129862529
34	Global	2006	Male	2.32508	31.13514	0.074677118
35	Global	2007	Female	2.49082	19.20596	0.129689993
36	Global	2007	Male	2.37066	31.55318	0.075132129
37	Global	2008	Female	2.4884	19.44514	0.127970538
38	Global	2008	Male	2.36882	32.02317	0.073972049
39	Global	2009	Female	2.47903	19.62791	0.126301091
40	Global	2009	Male	2.36247	32.38969	0.072938946
41	Global	2010	Female	2.49546	19.70498	0.126641222
42	Global	2010	Male	2.39794	32.57933	0.073603261
43	Global	2011	Female	2.50583	19.67164	0.127382901
44	Global	2011	Male	2.42104	32.59384	0.074279068
45	Global	2012	Female	2.5217	19.69293	0.12805092
46	Global	2012	Male	2.43446	32.70533	0.074436259
47	Global	2013	Female	2.52609	19.70623	0.128187494
48	Global	2013	Male	2.4664	32.79371	0.075209429
49	Global	2014	Female	2.5193	19.67876	0.128021206
50	Global	2014	Male	2.47969	32.80869	0.075580236
51	Global	2015	Female	2.51518	19.63939	0.128068248
52	Global	2015	Male	2.49246	32.79619	0.075998327
53	Global	2016	Female	2.49142	19.54959	0.127441166
54	Global	2016	Male	2.47486	32.68026	0.075729573
55	Global	2017	Female	2.45171	19.48114	0.125850618
56	Global	2017	Male	2.45553	32.57551	0.075379575
57	Global	2018	Female	2.40532	19.41337	0.12390011
58	Global	2018	Male	2.41978	32.45193	0.074565164

59	Global	2019	Female	2.34396	19.31766	0.12133765
60	Global	2019	Male	2.38116	32.28673	0.07375037
61	Global	2020	Female	2.28856	19.11617	0.119718476
62	Global	2020	Male	2.3448	31.98871	0.073300715
63	Global	2021	Female	2.24758	18.85161	0.11922479
64	Global	2021	Male	2.32127	31.63898	0.073367386
65	High SDI	1990	Female	4.66282	36.02974	0.129415812
66	High SDI	1990	Male	5.31262	65.12216	0.081579324
67	High SDI	1991	Female	4.72328	36.01441	0.131149582
68	High SDI	1991	Male	5.38722	64.92722	0.082973147
69	High SDI	1992	Female	4.75685	36.04153	0.131982466
70	High SDI	1992	Male	5.43988	64.79637	0.083953498
71	High SDI	1993	Female	4.81295	36.10573	0.1333016
72	High SDI	1993	Male	5.46604	64.72281	0.08445304
73	High SDI	1994	Female	4.70486	36.18587	0.130019345
74	High SDI	1994	Male	5.34013	64.67506	0.08256865
75	High SDI	1995	Female	4.59686	36.27999	0.126705226
76	High SDI	1995	Male	5.2197	64.68511	0.080693943
77	High SDI	1996	Female	4.56666	36.51355	0.125067538
78	High SDI	1996	Male	5.15029	65.1163	0.079093646
79	High SDI	1997	Female	4.51073	36.92747	0.122150987
80	High SDI	1997	Male	5.036	66.08823	0.076201128
81	High SDI	1998	Female	4.45006	37.40629	0.118965456
82	High SDI	1998	Male	4.9629	67.24454	0.073803768
83	High SDI	1999	Female	4.24918	37.82849	0.112327407
84	High SDI	1999	Male	4.6952	68.20497	0.068839602
85	High SDI	2000	Female	4.21875	38.13183	0.11063597
86	High SDI	2000	Male	4.62109	68.71164	0.06725337
87	High SDI	2001	Female	4.18168	38.46013	0.108727539
88	High SDI	2001	Male	4.54182	68.93294	0.065887519
89	High SDI	2002	Female	4.17002	38.97356	0.106996207
90	High SDI	2002	Male	4.49694	69.28879	0.064901479
91	High SDI	2003	Female	4.1316	39.57763	0.104392251
92	High SDI	2003	Male	4.46992	69.78957	0.064048474
93	High SDI	2004	Female	4.07309	40.1495	0.101448067
94	High SDI	2004	Male	4.37857	70.33801	0.062250478
95	High SDI	2005	Female	4.09294	40.59969	0.100812139
96	High SDI	2005	Male	4.3929	70.95341	0.061912444
97	High SDI	2006	Female	4.09845	41.02566	0.099899776
98	High SDI	2006	Male	4.43645	71.72528	0.061853394
99	High SDI	2007	Female	4.14074	41.58782	0.099566171
100	High SDI	2007	Male	4.52617	72.71494	0.062245409
101	High SDI	2008	Female	4.1369	42.1792	0.098079182
102	High SDI	2008	Male	4.49932	73.7528	0.061005478
103	High SDI	2009	Female	4.1434	42.67695	0.097087442
104	High SDI	2009	Male	4.49314	74.59544	0.060233436
105	High SDI	2010	Female	4.17898	42.98673	0.097215641

106	High SDI	2010	Male	4.55613	75.13681	0.06063781
107	High SDI	2011	Female	4.20307	43.11434	0.097486598
108	High SDI	2011	Male	4.61303	75.41025	0.061172465
109	High SDI	2012	Female	4.24555	43.21278	0.098247547
110	High SDI	2012	Male	4.65914	75.61543	0.061616203
111	High SDI	2013	Female	4.24915	43.27205	0.098196286
112	High SDI	2013	Male	4.70682	75.7527	0.062134084
113	High SDI	2014	Female	4.22989	43.30219	0.097683061
114	High SDI	2014	Male	4.68045	75.82101	0.061730191
115	High SDI	2015	Female	4.22872	43.34309	0.097563812
116	High SDI	2015	Male	4.68975	75.91198	0.06177884
117	High SDI	2016	Female	4.21299	43.39592	0.097082519
118	High SDI	2016	Male	4.65597	76.0434	0.061227812
119	High SDI	2017	Female	4.1386	43.44661	0.09525722
120	High SDI	2017	Male	4.60045	76.18078	0.060388572
121	High SDI	2018	Female	4.07679	43.49728	0.093725058
122	High SDI	2018	Male	4.52573	76.27094	0.059337576
123	High SDI	2019	Female	3.98179	43.52879	0.091474826
124	High SDI	2019	Male	4.45669	76.24695	0.058450668
125	High SDI	2020	Female	3.87805	43.40532	0.089344968
126	High SDI	2020	Male	4.35522	75.84121	0.057425461
127	High SDI	2021	Female	3.84414	42.79468	0.089827593
128	High SDI	2021	Male	4.32998	75.18593	0.057590361
129	High-midd	1990	Female	1.42138	18.09183	0.078564602
130	High-midd	1990	Male	1.55005	35.91339	0.04316078
131	High-midd	1991	Female	1.45797	18.13298	0.080404325
132	High-midd	1991	Male	1.58325	35.98058	0.044002837
133	High-midd	1992	Female	1.49376	18.20687	0.082043598
134	High-midd	1992	Male	1.62407	36.06081	0.045037103
135	High-midd	1993	Female	1.55459	18.299	0.084955156
136	High-midd	1993	Male	1.68582	36.12044	0.046672204
137	High-midd	1994	Female	1.59176	18.38585	0.086575209
138	High-midd	1994	Male	1.7151	36.11388	0.047491303
139	High-midd	1995	Female	1.62169	18.45096	0.087892019
140	High-midd	1995	Male	1.75464	36.04847	0.048674599
141	High-midd	1996	Female	1.63257	18.52515	0.088127403
142	High-midd	1996	Male	1.75464	35.93683	0.04882563
143	High-midd	1997	Female	1.63161	18.64465	0.087511045
144	High-midd	1997	Male	1.73916	35.83603	0.048530932
145	High-midd	1998	Female	1.66494	18.78962	0.088609816
146	High-midd	1998	Male	1.7516	35.74787	0.04899881
147	High-midd	1999	Female	1.66413	18.92296	0.087942478
148	High-midd	1999	Male	1.76829	35.65047	0.04960084
149	High-midd	2000	Female	1.71162	19.01967	0.08999227
150	High-midd	2000	Male	1.80425	35.58927	0.050696454
151	High-midd	2001	Female	1.73829	19.12648	0.090883723
152	High-midd	2001	Male	1.83355	35.58695	0.051523064

153	High-midd	2002	Female	1.77129	19.33591	0.091606188
154	High-midd	2002	Male	1.87128	35.7426	0.052354434
155	High-midd	2003	Female	1.74573	19.61534	0.088998324
156	High-midd	2003	Male	1.86514	35.98811	0.051826518
157	High-midd	2004	Female	1.72	19.88847	0.086482171
158	High-midd	2004	Male	1.84914	36.21612	0.051058548
159	High-midd	2005	Female	1.71567	20.10833	0.085321224
160	High-midd	2005	Male	1.83668	36.41131	0.050442457
161	High-midd	2006	Female	1.69408	20.29726	0.083463729
162	High-midd	2006	Male	1.80024	36.61149	0.049171399
163	High-midd	2007	Female	1.72971	20.57943	0.084050596
164	High-midd	2007	Male	1.83071	37.03503	0.049431852
165	High-midd	2008	Female	1.74387	20.90966	0.083400084
166	High-midd	2008	Male	1.84754	37.53733	0.049218787
167	High-midd	2009	Female	1.75217	21.17431	0.082749823
168	High-midd	2009	Male	1.85021	37.92788	0.048782314
169	High-midd	2010	Female	1.78148	21.33175	0.083512987
170	High-midd	2010	Male	1.89082	38.14068	0.049574895
171	High-midd	2011	Female	1.80177	21.37821	0.084280491
172	High-midd	2011	Male	1.90012	38.18962	0.049754886
173	High-midd	2012	Female	1.80711	21.48009	0.084129327
174	High-midd	2012	Male	1.91775	38.39314	0.049950328
175	High-midd	2013	Female	1.80579	21.59334	0.08362713
176	High-midd	2013	Male	1.9149	38.61648	0.049587571
177	High-midd	2014	Female	1.81984	21.61683	0.084186252
178	High-midd	2014	Male	1.92223	38.6875	0.049686067
179	High-midd	2015	Female	1.82697	21.57473	0.084680884
180	High-midd	2015	Male	1.9551	38.6366	0.050602256
181	High-midd	2016	Female	1.77787	21.41139	0.083033686
182	High-midd	2016	Male	1.93007	38.37871	0.05029018
183	High-midd	2017	Female	1.75146	21.21042	0.082575671
184	High-midd	2017	Male	1.91448	38.07197	0.050285925
185	High-midd	2018	Female	1.70938	21.01571	0.081337978
186	High-midd	2018	Male	1.89269	37.76384	0.050119175
187	High-midd	2019	Female	1.66576	20.8342	0.079953362
188	High-midd	2019	Male	1.84785	37.48858	0.049290998
189	High-midd	2020	Female	1.6459	20.60245	0.079888614
190	High-midd	2020	Male	1.8175	37.19041	0.048870151
191	High-midd	2021	Female	1.57655	20.4481	0.077100013
192	High-midd	2021	Male	1.73592	36.67886	0.047327403
193	Low SDI	1990	Female	1.60628	3.609416	0.445025612
194	Low SDI	1990	Male	1.37351	3.944669	0.348192869
195	Low SDI	1991	Female	1.61081	3.594021	0.448190515
196	Low SDI	1991	Male	1.37786	3.935707	0.350091222
197	Low SDI	1992	Female	1.61803	3.579296	0.452051769
198	Low SDI	1992	Male	1.37806	3.929674	0.350681697
199	Low SDI	1993	Female	1.62206	3.566333	0.454824685

200	Low SDI	1993	Male	1.38406	3.926495	0.352492998
201	Low SDI	1994	Female	1.64732	3.554435	0.463455265
202	Low SDI	1994	Male	1.40533	3.925835	0.357969798
203	Low SDI	1995	Female	1.66319	3.54432	0.469254725
204	Low SDI	1995	Male	1.40618	3.928152	0.357975804
205	Low SDI	1996	Female	1.62922	3.534675	0.460925779
206	Low SDI	1996	Male	1.38871	3.932596	0.353129256
207	Low SDI	1997	Female	1.60053	3.523268	0.454275075
208	Low SDI	1997	Male	1.379	3.940922	0.349919306
209	Low SDI	1998	Female	1.57274	3.513074	0.447682655
210	Low SDI	1998	Male	1.38265	3.950752	0.349972333
211	Low SDI	1999	Female	1.53945	3.505613	0.439139739
212	Low SDI	1999	Male	1.35889	3.961047	0.343063653
213	Low SDI	2000	Female	1.52268	3.501961	0.434806491
214	Low SDI	2000	Male	1.35003	3.971534	0.339926607
215	Low SDI	2001	Female	1.5126	3.502774	0.431829061
216	Low SDI	2001	Male	1.33006	3.982922	0.333941806
217	Low SDI	2002	Female	1.52277	3.503047	0.434697378
218	Low SDI	2002	Male	1.32007	3.997759	0.330202058
219	Low SDI	2003	Female	1.51029	3.504681	0.430933845
220	Low SDI	2003	Male	1.33626	4.014134	0.33288863
221	Low SDI	2004	Female	1.48154	3.507317	0.42241385
222	Low SDI	2004	Male	1.32982	4.030985	0.329898946
223	Low SDI	2005	Female	1.46084	3.509488	0.416254136
224	Low SDI	2005	Male	1.30737	4.049268	0.3228666
225	Low SDI	2006	Female	1.46492	3.515628	0.416687361
226	Low SDI	2006	Male	1.31377	4.072942	0.32256156
227	Low SDI	2007	Female	1.46185	3.522694	0.414979443
228	Low SDI	2007	Male	1.32374	4.100644	0.322813611
229	Low SDI	2008	Female	1.46465	3.531457	0.414743079
230	Low SDI	2008	Male	1.3291	4.128758	0.321913138
231	Low SDI	2009	Female	1.46148	3.540295	0.412814083
232	Low SDI	2009	Male	1.33316	4.154855	0.320867237
233	Low SDI	2010	Female	1.45906	3.548078	0.411225331
234	Low SDI	2010	Male	1.34917	4.17554	0.323112971
235	Low SDI	2011	Female	1.479	3.56048	0.415394308
236	Low SDI	2011	Male	1.35955	4.195357	0.32405963
237	Low SDI	2012	Female	1.53663	3.576298	0.429670208
238	Low SDI	2012	Male	1.35959	4.218287	0.322308866
239	Low SDI	2013	Female	1.63354	3.593767	0.454548155
240	Low SDI	2013	Male	1.42823	4.238425	0.336971966
241	Low SDI	2014	Female	1.66692	3.611279	0.461588179
242	Low SDI	2014	Male	1.47433	4.253169	0.346642083
243	Low SDI	2015	Female	1.63348	3.627586	0.450295152
244	Low SDI	2015	Male	1.4806	4.261661	0.347422191
245	Low SDI	2016	Female	1.61539	3.65211	0.442317076
246	Low SDI	2016	Male	1.48386	4.262434	0.348125219

247	Low SDI	2017	Female	1.61363	3.683421	0.438079156
248	Low SDI	2017	Male	1.49836	4.258044	0.351888192
249	Low SDI	2018	Female	1.59509	3.713179	0.429575422
250	Low SDI	2018	Male	1.46136	4.253718	0.343548467
251	Low SDI	2019	Female	1.58357	3.733004	0.424207415
252	Low SDI	2019	Male	1.47	4.254319	0.345531349
253	Low SDI	2020	Female	1.56846	3.736086	0.419814123
254	Low SDI	2020	Male	1.47787	4.321874	0.341951536
255	Low SDI	2021	Female	1.56015	3.759716	0.414965477
256	Low SDI	2021	Male	1.4509	4.332077	0.334920861
257	Low-middl	1990	Female	1.17492	5.342589	0.219915038
258	Low-middl	1990	Male	1.02918	6.496779	0.158413454
259	Low-middl	1991	Female	1.16657	5.346327	0.218201111
260	Low-middl	1991	Male	1.03105	6.487986	0.158916101
261	Low-middl	1992	Female	1.18642	5.348196	0.221835497
262	Low-middl	1992	Male	1.03957	6.484466	0.160317412
263	Low-middl	1993	Female	1.18325	5.348614	0.221225872
264	Low-middl	1993	Male	1.04941	6.489702	0.161703689
265	Low-middl	1994	Female	1.18615	5.346301	0.22186286
266	Low-middl	1994	Male	1.05315	6.499849	0.162026335
267	Low-middl	1995	Female	1.21862	5.342157	0.228113654
268	Low-middl	1995	Male	1.0708	6.514855	0.164362964
269	Low-middl	1996	Female	1.21248	5.336142	0.227221257
270	Low-middl	1996	Male	1.07986	6.545514	0.164976533
271	Low-middl	1997	Female	1.19058	5.32799	0.223457745
272	Low-middl	1997	Male	1.08988	6.588679	0.165417199
273	Low-middl	1998	Female	1.19511	5.320902	0.224606767
274	Low-middl	1998	Male	1.09953	6.640608	0.165576449
275	Low-middl	1999	Female	1.17218	5.315443	0.220524418
276	Low-middl	1999	Male	1.07188	6.687112	0.160290291
277	Low-middl	2000	Female	1.15333	5.314779	0.217003458
278	Low-middl	2000	Male	1.07183	6.723274	0.159420744
279	Low-middl	2001	Female	1.15363	5.3218	0.216774967
280	Low-middl	2001	Male	1.08834	6.758389	0.161035464
281	Low-middl	2002	Female	1.16741	5.33444	0.218843553
282	Low-middl	2002	Male	1.09239	6.797787	0.160697333
283	Low-middl	2003	Female	1.17133	5.350174	0.218933849
284	Low-middl	2003	Male	1.12008	6.839693	0.163762048
285	Low-middl	2004	Female	1.16075	5.365785	0.216324602
286	Low-middl	2004	Male	1.1286	6.8787	0.164071959
287	Low-middl	2005	Female	1.16232	5.379911	0.216048254
288	Low-middl	2005	Male	1.11778	6.916708	0.161605202
289	Low-middl	2006	Female	1.17932	5.394135	0.218630293
290	Low-middl	2006	Male	1.12159	6.963028	0.161078154
291	Low-middl	2007	Female	1.19597	5.412098	0.220981522
292	Low-middl	2007	Male	1.14739	7.025939	0.163307008
293	Low-middl	2008	Female	1.20349	5.43172	0.221566313

294	Low-middl	2008	Male	1.16311	7.096926	0.16388887
295	Low-middl	2009	Female	1.18196	5.451908	0.216797629
296	Low-middl	2009	Male	1.15681	7.165509	0.161441172
297	Low-middl	2010	Female	1.17261	5.470834	0.21433867
298	Low-middl	2010	Male	1.16376	7.226721	0.161035111
299	Low-middl	2011	Female	1.17784	5.491853	0.214470454
300	Low-middl	2011	Male	1.16687	7.289346	0.160079467
301	Low-middl	2012	Female	1.1914	5.520177	0.215826798
302	Low-middl	2012	Male	1.14792	7.364907	0.155863826
303	Low-middl	2013	Female	1.23865	5.553019	0.223059724
304	Low-middl	2013	Male	1.20202	7.443597	0.161483952
305	Low-middl	2014	Female	1.26993	5.588479	0.227240587
306	Low-middl	2014	Male	1.26692	7.515296	0.168578939
307	Low-middl	2015	Female	1.26314	5.622516	0.224657387
308	Low-middl	2015	Male	1.23846	7.570288	0.163594776
309	Low-middl	2016	Female	1.26987	5.671039	0.223922161
310	Low-middl	2016	Male	1.22049	7.60815	0.160418308
311	Low-middl	2017	Female	1.29032	5.74037	0.224780253
312	Low-middl	2017	Male	1.24572	7.642681	0.162995188
313	Low-middl	2018	Female	1.29401	5.809429	0.222743813
314	Low-middl	2018	Male	1.25973	7.677183	0.164087662
315	Low-middl	2019	Female	1.29386	5.857335	0.220896269
316	Low-middl	2019	Male	1.26752	7.714437	0.164305249
317	Low-middl	2020	Female	1.28337	5.856395	0.219139142
318	Low-middl	2020	Male	1.26616	7.824082	0.161828606
319	Low-middl	2021	Female	1.26853	5.902534	0.214913068
320	Low-middl	2021	Male	1.2615	7.872348	0.160244548
321	Middle SD	1990	Female	0.7829	6.817655	0.114833933
322	Middle SD	1990	Male	0.80771	11.11321	0.072679856
323	Middle SD	1991	Female	0.77555	6.846698	0.113273068
324	Middle SD	1991	Male	0.81801	11.1267	0.073517842
325	Middle SD	1992	Female	0.79037	6.873716	0.114984037
326	Middle SD	1992	Male	0.82464	11.1489	0.073966478
327	Middle SD	1993	Female	0.78561	6.897425	0.113899417
328	Middle SD	1993	Male	0.82857	11.1784	0.074122207
329	Middle SD	1994	Female	0.78959	6.915872	0.1141714
330	Middle SD	1994	Male	0.81989	11.21405	0.073113124
331	Middle SD	1995	Female	0.79163	6.928809	0.114252514
332	Middle SD	1995	Male	0.81284	11.25569	0.072216076
333	Middle SD	1996	Female	0.77833	6.937689	0.11218876
334	Middle SD	1996	Male	0.80205	11.31481	0.070885013
335	Middle SD	1997	Female	0.75147	6.946872	0.10817362
336	Middle SD	1997	Male	0.7827	11.39866	0.068666182
337	Middle SD	1998	Female	0.73952	6.958048	0.106282768
338	Middle SD	1998	Male	0.77635	11.49391	0.06754414
339	Middle SD	1999	Female	0.73608	6.972328	0.105571275
340	Middle SD	1999	Male	0.76232	11.58564	0.065798467

341	Middle SD	2000	Female	0.73738	6.992842	0.105447532
342	Middle SD	2000	Male	0.75599	11.65863	0.064844162
343	Middle SD	2001	Female	0.73154	7.024523	0.104140237
344	Middle SD	2001	Male	0.76238	11.73125	0.064987444
345	Middle SD	2002	Female	0.74322	7.066408	0.105175967
346	Middle SD	2002	Male	0.75765	11.81845	0.06410725
347	Middle SD	2003	Female	0.73667	7.111546	0.103587958
348	Middle SD	2003	Male	0.77416	11.90892	0.06500713
349	Middle SD	2004	Female	0.7345	7.154605	0.102660871
350	Middle SD	2004	Male	0.78466	11.99383	0.065421919
351	Middle SD	2005	Female	0.72799	7.195227	0.101176616
352	Middle SD	2005	Male	0.77132	12.06498	0.063930488
353	Middle SD	2006	Female	0.73142	7.274312	0.100548781
354	Middle SD	2006	Male	0.7641	12.23748	0.062439227
355	Middle SD	2007	Female	0.7279	7.409781	0.098234698
356	Middle SD	2007	Male	0.77068	12.5591	0.061364503
357	Middle SD	2008	Female	0.72597	7.562949	0.095989814
358	Middle SD	2008	Male	0.76915	12.92946	0.059488246
359	Middle SD	2009	Female	0.70835	7.702979	0.09195746
360	Middle SD	2009	Male	0.75712	13.25779	0.057107929
361	Middle SD	2010	Female	0.70221	7.785356	0.09019644
362	Middle SD	2010	Male	0.75304	13.43099	0.056067286
363	Middle SD	2011	Female	0.70174	7.834189	0.089573647
364	Middle SD	2011	Male	0.75289	13.52268	0.05567588
365	Middle SD	2012	Female	0.70863	7.883175	0.089892011
366	Middle SD	2012	Male	0.74456	13.62393	0.054650535
367	Middle SD	2013	Female	0.72866	7.932438	0.09185808
368	Middle SD	2013	Male	0.77096	13.72614	0.056167056
369	Middle SD	2014	Female	0.73616	7.992895	0.092101444
370	Middle SD	2014	Male	0.79643	13.84147	0.057539663
371	Middle SD	2015	Female	0.74536	8.042658	0.092676368
372	Middle SD	2015	Male	0.80227	13.91593	0.057651135
373	Middle SD	2016	Female	0.75141	8.115485	0.092590133
374	Middle SD	2016	Male	0.8074	13.99944	0.057673856
375	Middle SD	2017	Female	0.75128	8.2051	0.09156265
376	Middle SD	2017	Male	0.8022	14.08485	0.056955059
377	Middle SD	2018	Female	0.7383	8.284454	0.089118963
378	Middle SD	2018	Male	0.79452	14.15544	0.056128205
379	Middle SD	2019	Female	0.73071	8.326608	0.087756005
380	Middle SD	2019	Male	0.78976	14.19703	0.055628688
381	Middle SD	2020	Female	0.72094	8.178413	0.088151044
382	Middle SD	2020	Male	0.79035	13.91834	0.056784518
383	Middle SD	2021	Female	0.71737	8.205239	0.087428851
384	Middle SD	2021	Male	0.78434	13.89911	0.056430689

ns
